# Developmentally distinct activities of the exocyst enable rapid cell elongation and determine meristem size during primary root growth in Arabidopsis

**DOI:** 10.1186/s12870-014-0386-0

**Published:** 2014-12-31

**Authors:** Rex A Cole, Samantha A McInally, John E Fowler

**Affiliations:** Botany and Plant Pathology, Oregon State University, 2082 Cordley Hall, Corvallis, 97331 OR USA

**Keywords:** Exocyst, Root growth, Meristem, Cell expansion, Auxin, Brassinosteroid

## Abstract

**Background:**

Exocytosis is integral to root growth: trafficking components of systems that control growth (e.g., PIN auxin transport proteins) to the plasma membrane, and secreting materials that expand the cell wall to the apoplast. Spatiotemporal regulation of exocytosis in eukaryotes often involves the exocyst, an octameric complex that tethers selected secretory vesicles to specific sites on the plasma membrane and facilitates their exocytosis. We evaluated Arabidopsis lines with mutations in four exocyst components (SEC5, SEC8, EXO70A1 and EXO84B) to explore exocyst function in primary root growth.

**Results:**

The mutants have root growth rates that are 82% to 11% of wild-type. Even in lines with the most severe defects, the organization of the quiescent center and tissue layers at the root tips appears similar to wild-type, although meristematic, transition, and elongation zones are shorter. Reduced cell production rates in the mutants are due to the shorter meristems, but not to lengthened cell cycles. Additionally, mutants demonstrate reduced anisotropic cell expansion in the elongation zone, but not the meristematic zone, resulting in shorter mature cells that are similar in shape to wild-type. As expected, hypersensitivity to brefeldin A links the mutant root growth defect to altered vesicular trafficking. Several experimental approaches (e.g., dose–response measurements, localization of signaling components) failed to identify aberrant auxin or brassinosteroid signaling as a primary driver for reduced root growth in exocyst mutants.

**Conclusions:**

The exocyst participates in two spatially distinct developmental processes, apparently by mechanisms not directly linked to auxin or brassinosteroid signaling pathways, to help establish root meristem size, and to facilitate rapid cell expansion in the elongation zone.

**Electronic supplementary material:**

The online version of this article (doi:10.1186/s12870-014-0386-0) contains supplementary material, which is available to authorized users.

## Background

Roots grow into a variety of challenging local environments from which they must obtain the water, nutrients, and anchorage essential for plant survival. The stem cells and meristem from which all root tissues and future growth will derive are located in a vulnerable position at the tip of the root. Shielded in this position by a multilayered root cap, the root meristem is continuously thrust by growth into the unknown and potentially damaging frontier of the plant’s soil environment. This root structure allows the meristem to be in close proximity to soil conditions so that it can optimally adjust root growth and development to meet the needs of the plant, while simultaneously responding to the specific demands of its local environment. Such plasticity of growth is achieved by modulating cellular development within a well-defined and robustly controlled root tip structure. In the root tips of Arabidopsis, cells divide regimentally to align in files; and within those files individual cells progressively alter their growth mechanisms, which at first support cell division, and then accelerated cell elongation, and finally cell differentiation and maturation, to form the root’s functionally diverse tissues. Fundamentally, growth of root cells requires controlled relaxation of their cell walls to facilitate expansion of particular cell surfaces, balanced by strengthening that ensures the integrity of cell walls will not be compromised during the expansion process. In response to these complex demands, complex networks of interacting and compensatory mechanisms have evolved to control, coordinate, and maintain primary root growth [1-7].

Within the intricate cellular infrastructure that supports root growth is the secretory system, by which proteins, lipid, and carbohydrates are packaged into membrane-bound secretory vesicles and delivered for secretion to the growing plasma membrane and cell wall [8]. Secretion involves exocytosis: the fusion of secretory vesicles with the plasma membrane and the expelling of vesicle contents into the apoplast. Exocytosis delivers growth-related membrane proteins to the plasma membrane, including receptors (e.g. the brassinosteroid receptor, BRI1 [9]), signaling proteins (e.g. CRK5 [10]), transporters (e.g. PIN auxin transport facilitators [11]), and proteins to build the cell wall (e.g. components of the cellulose synthase complex [12]). Equally important to root growth is the secretion to the apoplast of hormones (e.g. brassinosteroids [13]), proteins that modify the cell wall (e.g. expansins [14]), and materials to build additional cell wall (e.g. pectins and hemicelluloses [15]). Consequently, the secretory system and the process of exocytosis are essential to both root growth and the control systems that regulate that growth.

In eukaryotes the spatio-temporal regulation of exocytosis for a number of developmental processes has been found to involve the exocyst, a complex of eight proteins. The eight proteins of the exocyst complex, SEC3, SEC5, SEC6, SEC8, SEC10, SEC15, EXO70, and EXO84, have a coiled coil structure, and the resultant protein rods ultimately assemble to form a tether between secretory vesicles and the plasma membrane prior to membrane fusion, which is mediated by SNARE proteins [16-19]. The molecular details of the exocyst’s role in exocytosis are incompletely understood, but attention has focused on two aspects of the exocyst’s tethering function: its role as a landmark specifying the site for exocytosis, and its role as a facilitator of exocytosis. The landmark function involves exocyst localization to specialized cortical domains at the plasma membrane, where key membrane components are enriched and poised for assembly into the machinery for exocytosis [20]. In plants, the components of the exocyst that are particularly important to this landmark function, and how the exocyst is localized to a specific site, are uncertain. However, both the formation of the cortical domains and tethering of vesicles by the exocyst are thought to be under the control of small GTPases and phosphoinositides, as they are in non-plant species [20,21]. The facilitator function of the exocyst provides increased rates of vesicle docking and fusion events, and it has been speculated that this may involve an exocyst role in localized SNARE assembly [20-23]. Supportive of such a speculation, an interaction between an exocyst component, EXO70B2, and SNARE protein, SNAP33, was identified in a yeast two-hybrid screen of Arabidopsis proteins [24]; similar interactions have been observed in *S. cerevisiae* [22]. The two functions of the exocyst, i.e. as a landmark or as an exocytosis facilitator, may be separable, as suggested by the observation that small GTPases appear to differentially regulate these two roles of the exocyst in non-plant species [21].

The exocyst functions as a complex in plants [19,25-27], where it is intimately associated with the process of growth. Mutation of exocyst components is associated with aberrant tip growth in pollen tubes [27,28], decreased polarized growth of root hairs [29], reduced elongation of hypocotyls in dark grown seedlings [27], dwarfism [29,30], altered root tracheary element development [31], and defects in cytokinesis [30,32,33]. Recently, the exocyst complex has been visualized in epidermal cells of the root meristematic, elongation, and maturation zones in Arabidopsis, demonstrating that subunits of the exocyst complex dynamically dock and undock at the plasma membrane, potentially creating sites for vesicle tethering and exocytosis [34,35]. In addition, the trafficking dynamics of the BRI1 brassinosteroid receptor and PIN auxin transporters in the root are altered in exocyst mutants, with the PIN trafficking defect thought to underlie the compromised polar auxin transport in mutant roots [36]. Another potential linkage of the exocyst and auxin is derived from characterization of a plasma membrane-localized scaffold protein, Interactor of Constitutive active ROP 1 (ICR1), which is required to maintain the primary root meristem [37]. ICR1 interacts with both small ROP GTPases and the exocyst subunit, SEC3, and also affects trafficking of PIN auxin transporters to and from the plasma membrane in Arabidopsis roots [37,38]. Thus, it is evident that the exocyst could play an important role in root growth, with current data pointing toward functions in auxin and/or brassinosteroid signaling [36,38].

We therefore sought to investigate the exocyst’s role within the integrated network of mechanisms that regulate and produce primary root growth in *Arabidopsis thaliana*, focusing on the hypothetical auxin- and brassinosteroid-related mechanisms. Seedlings with T-DNA insert mutations in various exocyst components that resulted in reductions, sometimes profound, in the primary root growth rate were evaluated. Surprisingly, previously demonstrated roles for the exocyst in cytokinesis, as well as PIN protein and BRI1 receptor trafficking, [30,33,36], did not appear to adequately explain the mutant root growth phenotype. However, a detailed analysis of various growth parameters revealed that exocyst mutants exhibited defects in both root cell production rates (arising from shorter meristems) and mature cell lengths (arising from slower rates of cell elongation), implicating the exocyst in these two distinct developmental processes, likely through distinctive mechanisms.

## Results

### Mutations in components of the exocyst result in dwarfism and a primary root growth defect

**Figure 1:**
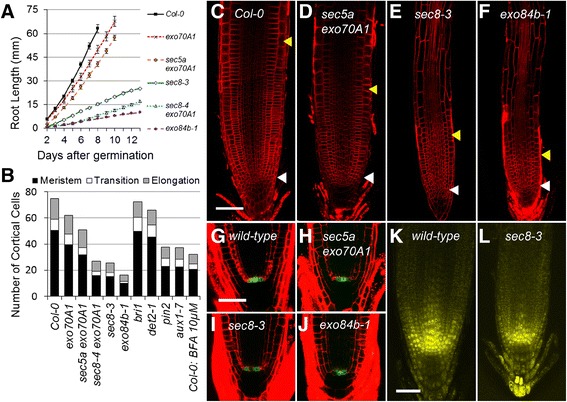
**Slower primary root growth in exocyst mutants is associated with shorter root growth zones. (A)** Root growth on vertical plates is slower in exocyst mutants than Col-0, with defects ranging from mild (e.g. *exo70A1-2*) to quite severe (e.g. *exo84b-1*) (n = 8–19 roots for each genotype; error bars represent standard error). **(B)** The number of cells in the meristem, transition, and elongation growth zones is reduced in exocyst mutants in correlation with the root growth defect. Brassinosteroid (*bri1* (SALK_003371), *det2-1*) and auxin transport (*pin2-1*, *aux1-7*) mutants, as well as brefeldin A (BFA)-treated roots are shown for comparison. Error bars for meristem and elongation zone data shown in Figure 2. **(C-L)** The shorter growth zones in exocyst mutants include shorter meristems that maintain a structure similar to wild-type. **(C-F)** Confocal images of 8 day old seedling root tips stained with propidium iodide; white triangles and yellow triangles mark the distal and proximal ends of the meristem (i.e. the MZ) (bar = 100 microns applies to **C-F**). **(G-J)** Confocal images of 8 day old propidium iodide-stained mutant roots **(H-J)** show expression of *pWOX5-GFP* restricted to the quiescent center and similar to wild-type **(G)** (bar = 50 microns). **(K-L)** Confocal images of root tips expressing PLT1-YFP driven by its native promoter in *sec8-3* **(L)** and a wild-type sibling **(K)** (bar = 50 microns).

In order to explore the role of the exocyst in primary root growth, Arabidopsis seedlings with *T-DNA* insertion mutations in genes encoding exocyst components were evaluated, including mutations in *SEC8*, *SEC5a*, *EXO70A1*, and *EXO84b*. A broad range of phenotypes associated with the *exo70A1-2* mutation has previously been described [29]. Many mutations in exocyst components do not result in a discernible single mutant phenotype (e.g., *sec5a*), presumably because there are multiple copies of genes encoding most of exocyst components (e.g., *SEC5b*) leading to functional redundancy. However, a *sec5a* mutation combined with the *exo70A1-2* mutation results in a synergistic defect in hypocotyl elongation [27], and the same combination shows a more severe root growth defect than the *exo70A1-2* mutant alone (Figure 1A). There are three *EXO84* paralogs in the Arabidopsis genome, but mutants of one of them, *exo84b*, are severely dwarfed with dramatically shorter roots [30]. SEC8 is a single copy gene, and T-DNA insertions in the 5′ end of the gene (*sec8-1* and *sec8-3*) result in a severe pollen defect and complete gametophytic sterility, whereas insertions in at the 3′ end (e.g. *sec8-4* and *sec8-6*) produce only mild phenotypes [28]. In order to characterize null exocyst mutations in the sporophyte, a construct containing the wild-type *SEC8* gene driven by the pollen-specific *LAT52* promoter was transformed into *sec8-1* and *sec8-3* heterozygous seedlings. The construct rescued the pollen defect in the *sec8* mutants, allowing generation of seedlings homozygous for the mutation, and these proved to be extremely dwarfed (Additional file 1: Figure S1). RT-PCR (data not shown) suggests that the *LAT52* promoter can drive low-level transcription in the sporophyte (as also shown by Van Damme, [39]), such that these *sec8-1* and *sec8-3* homozygous lines probably do not represent complete nulls for SEC8. (For brevity, these *LAT52:: SEC8 sec8* lines will be henceforth referred to merely as *sec8-1* or *sec8-3* lines.) Additional lines were generated by combining the *sec8-4* or *sec8-6* mutations, which do not have an obvious phenotype in the sporophyte, with the *exo70A1-2* mutation. These combinations also synergistically inhibit hypocotyl elongation [27], and result in a severe dwarfism of the same order of magnitude as the *sec8-3* line. Notably, the various exocyst mutants and mutant combinations reduce plant growth by differing, characteristic amounts (Additional file 1: Figure S1).

**Figure 2:**
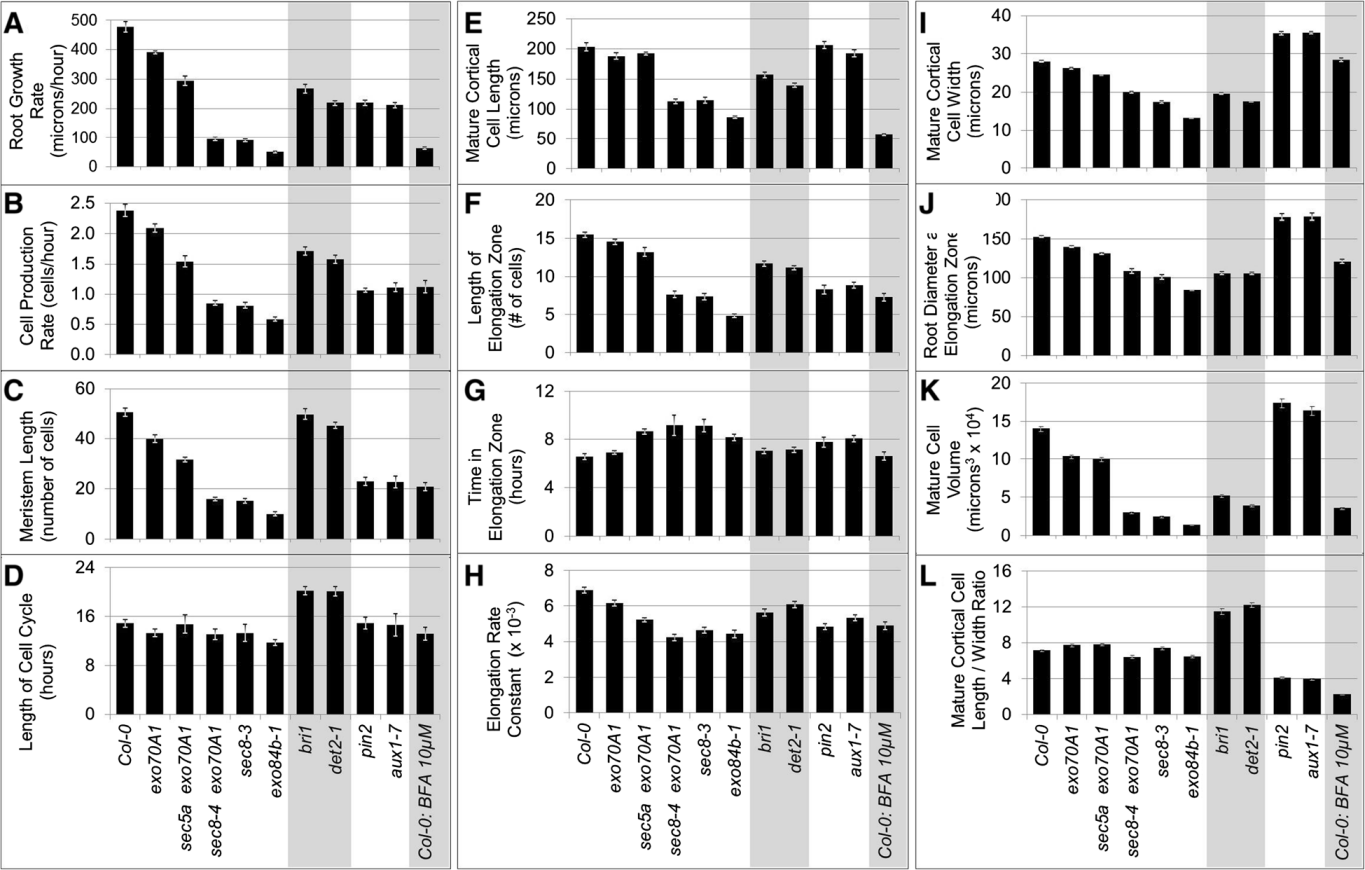
**Primary root growth characteristics in exocyst mutants. (A-L)** Characteristics of root growth in exocyst mutants are compared to Col-0 and several brassinosteroid and auxin mutants, as well as to BFA-treated Col-0 roots of 7 day old seedlings. Data for **B-L** represent averages for 14 cortical cell files (seven roots) evaluated from confocal images. Statistical comparisons to Col-0 (t-tests) are in Additional file 1: Figure S3. Error bars = standard error.

The dwarfism in seedlings with mutations in exocyst components includes shorter roots due to slower root growth rates, rather than premature termination of growth (Figure 1A). Mutant lines with T-DNA insertions in four different exocyst components demonstrate a dramatically wide range of primary root growth rates, which vary from a low of 52 microns/hour in *exo84b-1* mutants to 391 microns/hour in *exo70A1-1* mutants, compared to 478 microns/hour in *Columbia 0* (wild-type) (Figure 2A). Primary root growth in these mutants occurs at a nearly constant rate when evaluated from five to eight days after germination. Our focus was on this developmental period, corresponding to the time when the early expansion of the meristem has ceased, and after which the meristem size remains virtually constant [7,40]. One explanation for the observed range in severity of the root growth defects is that the distinct mutant combinations represent an allelic series of sorts, with each reducing exocyst complex function in a quantitative manner, which is subsequently manifested in quantitative effects on root growth rate. Thus, evaluating this set of mutants provides a potentially sensitive analysis for subtle effects of loss of exocyst function, i.e. small differences in the mutants can be considered more credible when the magnitude of those differences consistently correlate with the severity of the root growth defect across all mutants evaluated.

### Root growth defects in exocyst mutants are associated with shorter growth zones

Confocal microscopic images of seven-day old roots were evaluated to provide a detailed description of the meristematic and cell elongation root growth defects in exocyst mutants (Figure 1 and Additional file 2). Specifically, growth parameters were determined by measuring cell lengths along cortical cell files from the stem cell initials near the quiescent center to the beginning of the differentiation/maturation zone in seven-day old roots (see Additional file 2). This region spans the meristematic zone (MZ) where cells are dividing, the transition zone (TZ) where cells are not dividing but continue to elongate at a slow rate, and the elongation zone (EZ) where cell elongation increases exponentially until mature cell length is achieved, and cells enter the differentiation zone [5,41]. The lengths of these three distinct growth zones (MZ, TZ, and EZ) were dramatically shorter in the exocyst mutants (Figure 1B, 2C, and 2F). Notably, the overall cell file structure of the root tip was maintained in all the exocyst mutants examined, such that the consequences of a defect in cytokinesis, previously identified in the severe *exo84b* mutant [30], were not clearly evident at the tissue/organ level. Additionally, the identity and organization of the quiescent center at the root tip was stably maintained in the mutants, as assayed by several lines of evidence. The wild-type expression pattern of a marker for quiescent center identity (the *WOX5* promoter-driven GFP construct, [42]) was not disturbed in even the most severe mutant (Figure 1G-J). The preservation of the stem cell niche, as well as the overall structure of the meristem, is associated with a gradient of the *PLETHORA* transcription factors, *PLT1* and *PLT2* [43]. In exocyst mutants, YFP-labeled PLT1 and PLT2 proteins driven by their native promoters showed nuclear localization and a gradient pattern of expression that was similar to that of wild-type seedlings (Figure 1K and L, and Additional file 1: Figure S2), but compressed, coincident with the smaller size of the root meristems of the mutants. Thus, although mutations in components of the exocyst result in shorter growth zones, the overall root tip structure and tissue patterning in the mutants, which originate during embryogenesis, are similar to wild-type. However, the sizes of the MZ, TZ and EZ are clearly sensitive to reduced exocyst function.

**Figure 3:**
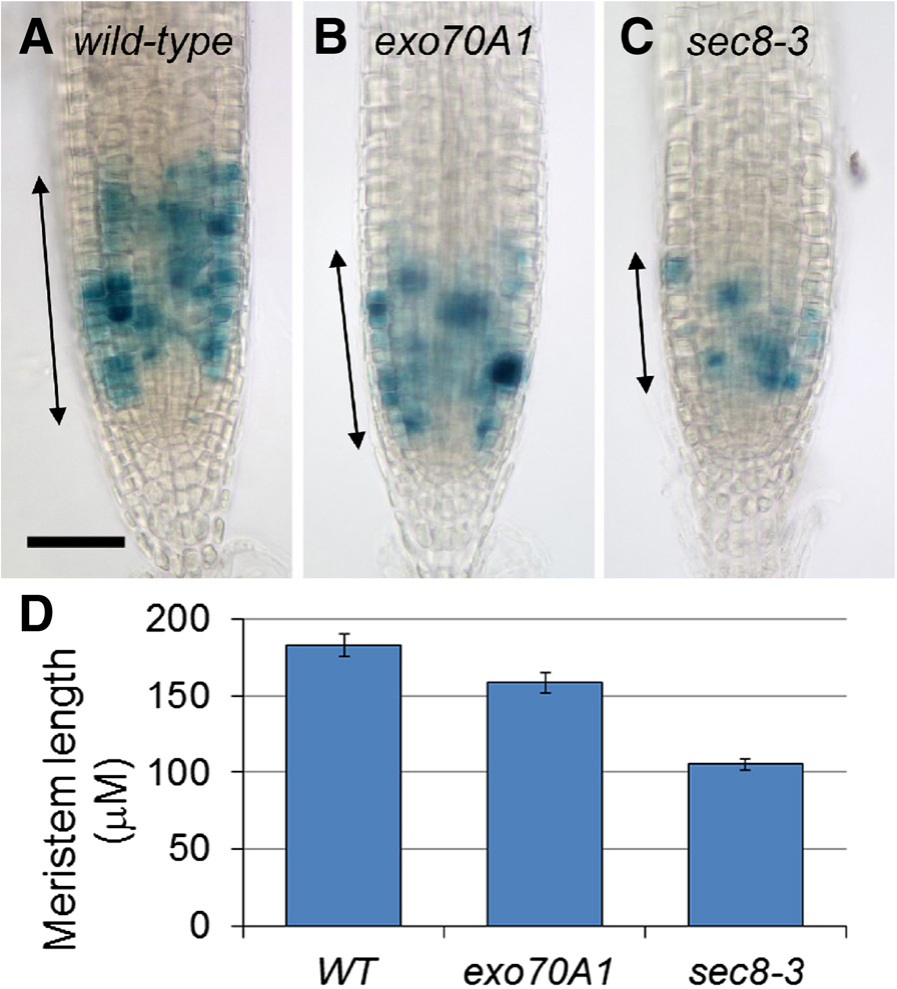
**Meristems are shorter in exocyst mutants.** *pCYCB:GUS* expression is a marker for cell division activity, and the size of the meristematic zone is designated by the span (arrows) over which GUS staining is detected, in wild-type **(A)**, exo70A1 **(B)**, and sec8-3 **(C)** root tips. Bar = 50 microns. **(D)** Average root meristem lengths measured by *CYCB:GUS* are significantly shorter in *exo70A1* (p < 0.01, *t*-test, n = 30) and *sec8-3* (p < 0.001, *t*-test, n = 19) mutants compared to wild-type siblings (n = 30).

The growth rate of plant roots depends upon the rate of cell production in the meristem, and the extent of anisotropic cell expansion in the root’s EZ. To initially evaluate whether exocyst mutations affect cell division patterns in the root meristem, a *CycB1::GUS* reporter was introduced into *sec8-3, exo70A1-1,* and *exo70A1-1 sec8-6* mutant lines. Fusion of the *CycB1* promoter with GUS and a mitotic degradation signal allows this reporter to mark only actively dividing cells [44]. As expected, GUS staining of the exocyst mutant roots revealed shorter meristems, associated with fewer dividing cells within this zone, compared to their wild-type siblings (p < 0.001, *t*-test, n > 22 roots, Figure 3). Analysis of confocal images of root cortical cell profiles from the MZ through the EZ was then used to estimate cell production rates and cell cycle lengths in this cell layer ([45], and see Methods). The roots of five different exocyst mutant lines (7 roots per mutant line) were studied in detail, representing a range of root growth rate defects: 11 percent of wild-type for *exo84b-1* to 82 percent of wild-type for *exo70A1-1*. Consistent with the *pCYCB::GUS* results, the exocyst mutants demonstrated a reduced cell production rate that correlated with the reduced root growth rate (Figures 2A and 2B). To determine if the reduced cell production rate was due to a slower rate of cell division, the average length of the cell cycle was estimated from the cell production rate and the number of cells in the MZ (Additional file 2; [46]). Brassinosteroid mutants, *bri1* and *det2-1*, known to have altered cell cycle progression and slower rates of cell division [47,48], served as controls and verified that our method was capable of detecting prolonged cell cycles. Surprisingly, the cell cycle length was not prolonged in the exocyst mutants (p > 0.05, *t*-test, n = 7), and is notably shorter than wild-type (i.e., more rapid cell division) in the most severe line, *exo84b-1* (Figure 2D). Thus, the reduced cell production rate associated with loss of exocyst function is largely due to a reduced number of dividing cells in the shorter MZ (Figure 2C), not slower cell division.

### The exocyst facilitates cell expansion in the elongation zone

**Figure 4:**
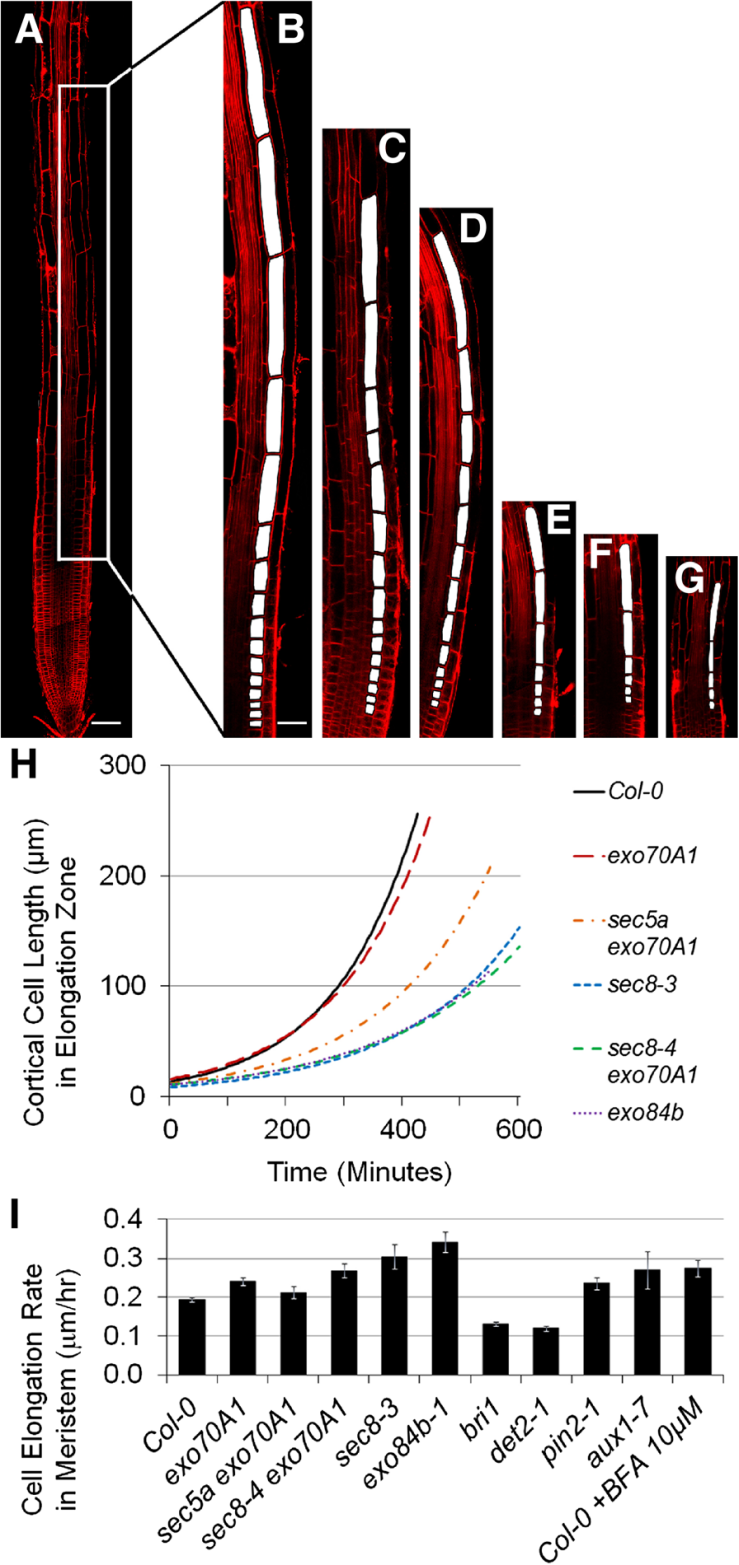
**Root cortical cells in exocyst mutants elongate at a slower rate in shorter elongation zones. (A)** Composite confocal image of a Col-0 root (bar = 100 microns). **(B-G)** Cortical cells in elongation zones, highlighted in white (bar = 50 μM). Col-0 **(A, B,)**; *exo70A1* **(C)**; *sec5a exo70A1* **(D)**; *sec8-4 exo70A1* **(E)**; *sec8-3* **(F)**; *exo84b-1* **(G). (H)** Exponential curves fitted to cell length data for elongation zones of 14 cell files (7 roots) of each genotype. The curves allow estimation of relative elongation rates for each genotype (Figure 2H) **(I)** Reduced cell elongation rates are not evident in the meristematic zones of exocyst mutants or Col-0 treated with 10 μM BFA compared to Col-0; error bars represent standard error with n = 7 roots per genotype. (see Additional file 2).

Cortical cell lengths were assessed to determine if a defect in cell elongation also contributed to the root growth defect in exocyst mutants. Mature cortical cell lengths, primarily the result of cell elongation in the EZ, were indeed reduced in the mutants with severe growth defects (Figure 2E, p < 0.001, *t*-test, n = 7). The reduced cell elongation in exocyst mutants could conceivably arise because cells in exocyst mutants have less time to elongate in their shorter elongation zones or because they elongate at a slower rate. To evaluate these two possibilities, data for the cortical cell length profiles in the EZ of fourteen cell files for each mutant genotype were analyzed to estimate elongation rates, as well as time spent in the EZ (detailed methods in Additional file 2). Exocyst mutants with severe root growth defects have significantly reduced rates of elongation compared to Col-0 (p < 0.001, Potthoff analysis) (Figure 4H and Additional file 2). Furthermore, except for the mild *exo70A1-1* mutant (which did not differ from wild-type), the time cortical cells of the exocyst mutants spent in the EZ was actually longer than that observed for wild-type Col-0 (Figure 2G, p < 0.01, *t*-test, n = 7). In other words, the reason mature cortical cells of exocyst mutants are shorter is not because they have spent less time elongating. Rather, loss of exocyst function is specifically associated with a slower rate of elongation in the EZ.

Mature cortical cell widths were also found to be significantly reduced in exocyst mutants (Figure 2I, p < 0.015, *t*-test, n = 7). The width reduction correlated with the severity of the root growth defect, and not surprisingly, with the width of the root (Figure 2J cell width measurements in the EZ). The cell width data, combined with data for mature cortical cell lengths, allow calculation of the average cell volume, as well as the average cell length-to-width ratio for each genotype (Figure 2K and 2L). The mature cortical cell volumes were dramatically reduced in the exocyst mutants, whereas the length-to-width ratio of mature cortical cells was only minimally altered compared to wild-type Col-0. Thus, the reductions in root growth rate in exocyst mutants are not exclusively associated with a reduction in mature cell lengths, but reflect a reduction in cell expansion in the EZ, resulting in mature cortical cells that achieve a near wild-type shape. Notably, cells of both brassinosteroid (*bri1, det2-1*) and auxin mutants (*pin2, aux1-7*) also alter cell expansion, but generate cells with aberrant cortical cell length/width ratios.

We were curious to know if the elongation defect in exocyst mutants was restricted to the elongation zone, or if it represented a more generalized defect (for example, a defect altering the basic structure and overall expansibility of the cell wall) that also influenced cell elongation in the meristem. To explore this question cortical cell lengths in the meristem were evaluated, and the rates of cell elongation in the meristem were estimated (Figure 4I and Additional file 2). The average cell elongation rates were determined to be slightly faster, not slower in the meristems of exocyst mutants compared to Col-0 (p < 0.05, *t*-test, n = 7), with the exception of *sec5a exo70A1-2*, in which the increase was not statistically significant. Thus, the reduced rate of root cell elongation in exocyst mutants occurs specifically in the EZ. A similar conclusion was reached when the width of cells along cell files in the MZ were measured and plotted as a function of cell position from the quiescent center. Cell widths expanded as the cells progressed shootward along the length of the meristem for Col-0 and all mutants evaluated (Additional file 2). This progression of cell width expansion in the exocyst mutants was essentially the same as Col-0, although there are fewer cells in the cell files of the shorter meristem in exocyst mutants. Thus, the role of the exocyst to enable root cell expansion (both length and width) appears to manifest primarily in the EZ.

In summary, reduced exocyst mutant root growth rates appear to be largely explained by a combination of 1) a reduced number of dividing cells in the meristem and 2) a reduced cell elongation rate in the EZ. This implies that the exocyst contributes to plant growth differentially during root development, functioning to achieve two apparently distinct outcomes: defining the sizes of the growth zones, and enabling rapid cellular expansion in the EZ.

### Root growth in exocyst mutants is hypersensitive to BFA treatment

**Figure 5:**
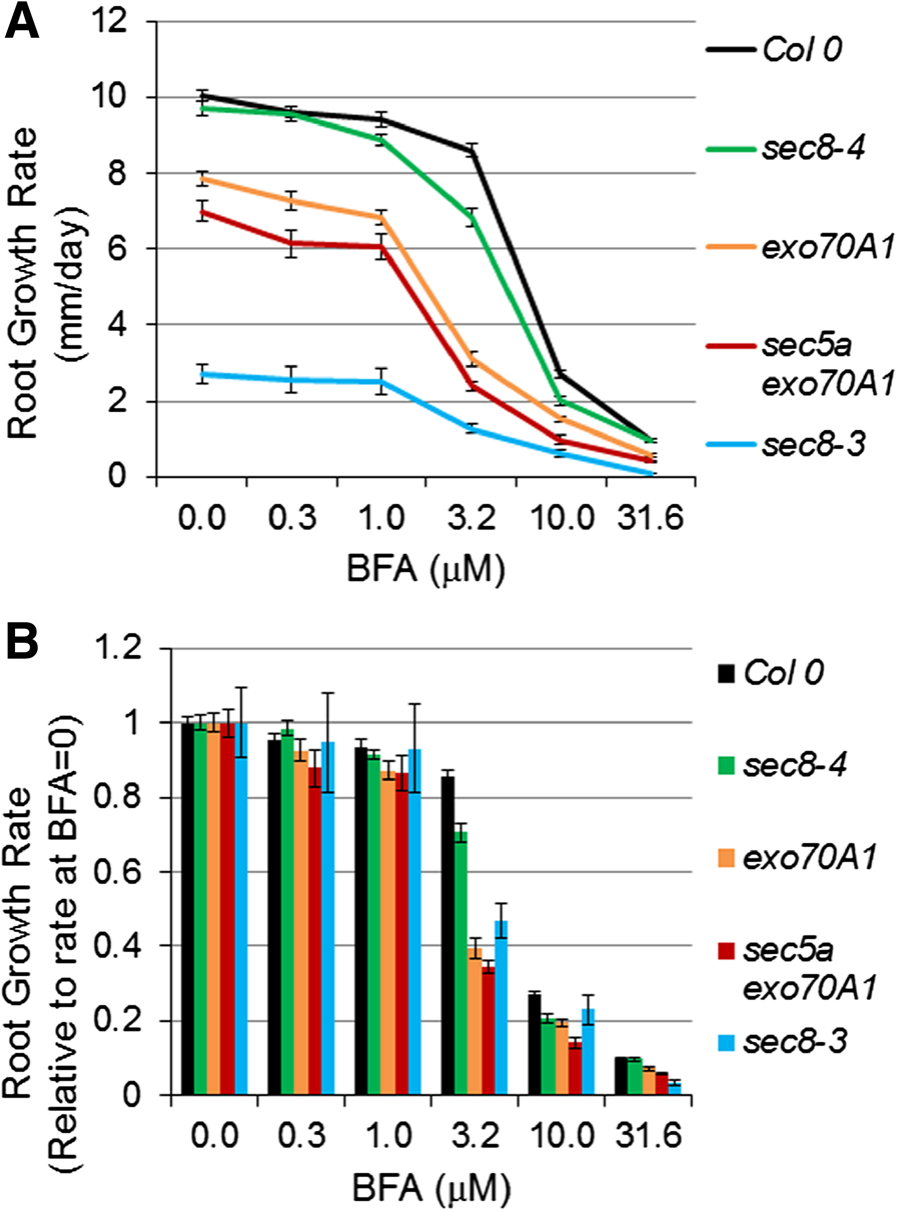
**Root growth rate response to brefeldin A (BFA). (A)** Exocyst mutants are hypersensitive to the root growth inhibiting effects of BFA at a concentration of 3.2 μM. **(B)** Root growth rate response to BFA normalized to the growth rate at BFA = 0. Normalized root growth rates of exocyst mutants are significantly lower than that of Col-0, at a BFA concentration of 3.2 μM (p < 0.001, *t*-test, n = 18-24). Bars = standard error.

To explore the hypothesis that the root growth defect in exocyst mutants reflected an altered secretory system, the sensitivity of root growth rate to the fungal toxin, brefeldin A (BFA) was assessed in exocyst mutants. BFA inhibits Golgi-based secretion and endocytic recycling, acting on guanine nucleotide exchange factors (e.g., GNOM) to alter the structure and function of the endomembrane system [49,50]. Alteration of secretion in BFA-treated plant cells is associated with altered delivery of polysaccharides and cell wall loosening factors to the cell wall [51-54], altered recycling of cell wall pectins in the root meristem [55], and a reduced root growth rate [56]. Although BFA is pleiotropic (e.g., it affects PIN protein recycling [56,57] as well as secretion of cell wall polysaccharides, and alters the root cell proteome accompanied by a remodeling of the actin cytoskeleton [58]), root growth hypersensitivity to BFA has been used as an indicator of a defect in vesicle transport and secretion [59]. Localization of exocyst subunits themselves to the plasma membrane is BFA-resistant [34,36], but root growth in exocyst mutants is hypersensitive to BFA (Figure 5), with the normalized growth rate reduced to a significantly greater extent in exocyst mutants compared to Col-0 (p < 0.001, *t*-test, n = 18-24) when exposed to 3.2 μM BFA. Such a result is consistent with a predicted function for the complex in vesicle trafficking to the PM, but does not rule out other functions given the pleiotropic action of BFA.

Roots of Col-0 seedlings grown on plates containing 10 μM BFA were evaluated to determine if they phenocopied the root growth defects associated with exocyst mutations. This concentration was chosen for comparison to the most severe exocyst mutants, as its effect on growth rate was similar to untreated *sec8-3* and *exo84b-1* lines. Measurements of cortical cell lengths indicated that the reduced growth rate in the BFA-treated Col-0 seedlings, like that of the exocyst mutants, was due to a reduced mature cell length and, to a lesser extent than in the comparable exocyst mutants, a reduced cell production rate (and associated shorter MZ) (Figure 2B, C, E, p < 0.001, *t*-test, n = 7). As with the exocyst mutants, the reduced mature cortical cell length was associated with a slower rate of elongation in the shorter EZ of BFA-treated Col-0 roots (Figure 2F, H, p < 0.001, *t*-test, n = 7). Remarkably, the exponential rate constant of this cortical cell elongation was nearly identical to that of the exocyst mutants with severe root growth defects (p > 0.05; Potthoff analysis; Additional file 2). Ultimately, the mature cortical cell lengths were shorter in the BFA-treated Col-0 roots than in the severe exocyst mutants, because the cells in the BFA-treated Col-0 roots spent less time in the EZ (Figure 2G). However, the BFA-treated mature cortical cell length-to-width ratio is quite distinct from that of the exocyst mutants (Figure 2L), likely reflecting the more pleiotropic nature of BFA action. The BFA results are consistent with the hypothesis that the exocyst’s function in root growth involves a role in secretory trafficking, but point toward some distinction: the exocyst appears more important for defining growth zone size.

### Altered auxin transport does not fully explain the root growth defect of exocyst mutants

Polarized auxin transport and an auxin gradient with a peak concentration at the quiescent center (QC) are key determinants of meristem structure and function, and thus root growth rate [42,60,61]. The size of the meristem depends in part upon an antagonistic interplay between auxin and cytokinin signaling, resulting in the shift from cell division to cell elongation at a particular location in the root tip [40,62-66]. Mutation of the exocyst component EXO70A1 results in a defect in acropetal (i.e., rootward) auxin transport and altered cycling of PIN auxin transport proteins to the plasma membrane in root epidermal cells [36]. We therefore hypothesized that reduced meristem lengths were observed in exocyst mutants because altered auxin transport shifted the auxin-cytokinin balance to favor a shift from cell division to elongation at a position closer to the root tip. To investigate this hypothesis, we measured primary root growth after attempting to shift the auxin-cytokinin balance in exocyst mutants and wild-type seedlings by growing them on media containing a series of concentrations of the native auxin: indole acetic acid (IAA), the synthetic auxin: 1-naphthaleneacetic acid (NAA), a cytokinin: N-6-benzyladenine, or the auxin transport inhibitor: naphthylphthalamic acid (NPA). Additionally, we measured the root growth response of exocyst mutants to 1-aminocyclopropanecarboxylic acid (ACC), an ethylene precursor. Ethylene promotes auxin biosynthesis and/or auxin transport to affect epidermal cell elongation and root growth [66-69], and the root growth response to ACC is altered in auxin-related mutants [70,71]. We reasoned that exocyst mutants should demonstrate altered sensitivity to these hormone manipulations if the reduced root growth rate in exocyst mutants is primarily the result of a defect in auxin transport.

**Figure 6:**
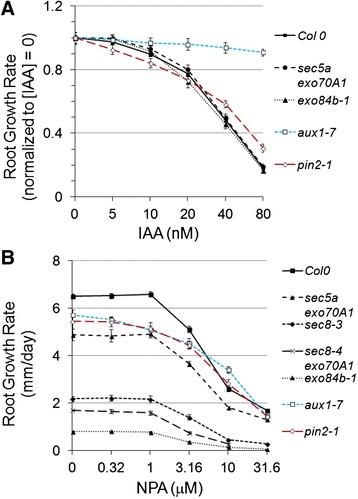
**The root growth response of exocyst mutants to IAA and NPA. (A)** IAA dose–response of exocyst mutant root growth was similar to Col-0, but different from auxin transport mutants *aux1-7* and *pin2-1*. **(B)** Exocyst mutants, *sec8-3* and *exo84b,* were slightly more sensitive to NPA at 3.16 and 10 μM compared to Col-0, whereas two auxin transport mutants were less sensitive.

Contrary to expectation, the root growth dose–response of exocyst mutants to exogenous auxins, cytokinin, or ACC proved not to be significantly different from that of wild-type Col-0 (Figure 6A, Additional file 1: Figure S4). The *pin2-1* mutant provided a contrasting control as, consistent with expectations, its sensitivity to IAA and NAA was distinct from wild-type. The contrast between exocyst and auxin mutant response was even more pronounced with NPA treatment (Figure 6B). Normalized root growth rates for severe exocyst mutants (i.e. *sec8-3, sec8-4 exo70A1-2,* and *exo84b-1*) were more sensitive than wild-type at NPA concentrations above 1 micromolar (p < 0.01, *t*-test, n = 20-28). However, the opposite response was exhibited by the *aux1-7* and *pin2-1* controls, which were less sensitive to NPA in the same concentration range (p < 0.01, *t*-test, n = 23-24). Thus, the response of exocyst mutant roots to exogenous hormone manipulation and auxin transport inhibition were not consistent with the hypothesis that exocyst-dependent PIN trafficking was the primary driver for the reduced root growth rate in exocyst mutants.

**Figure 7:**
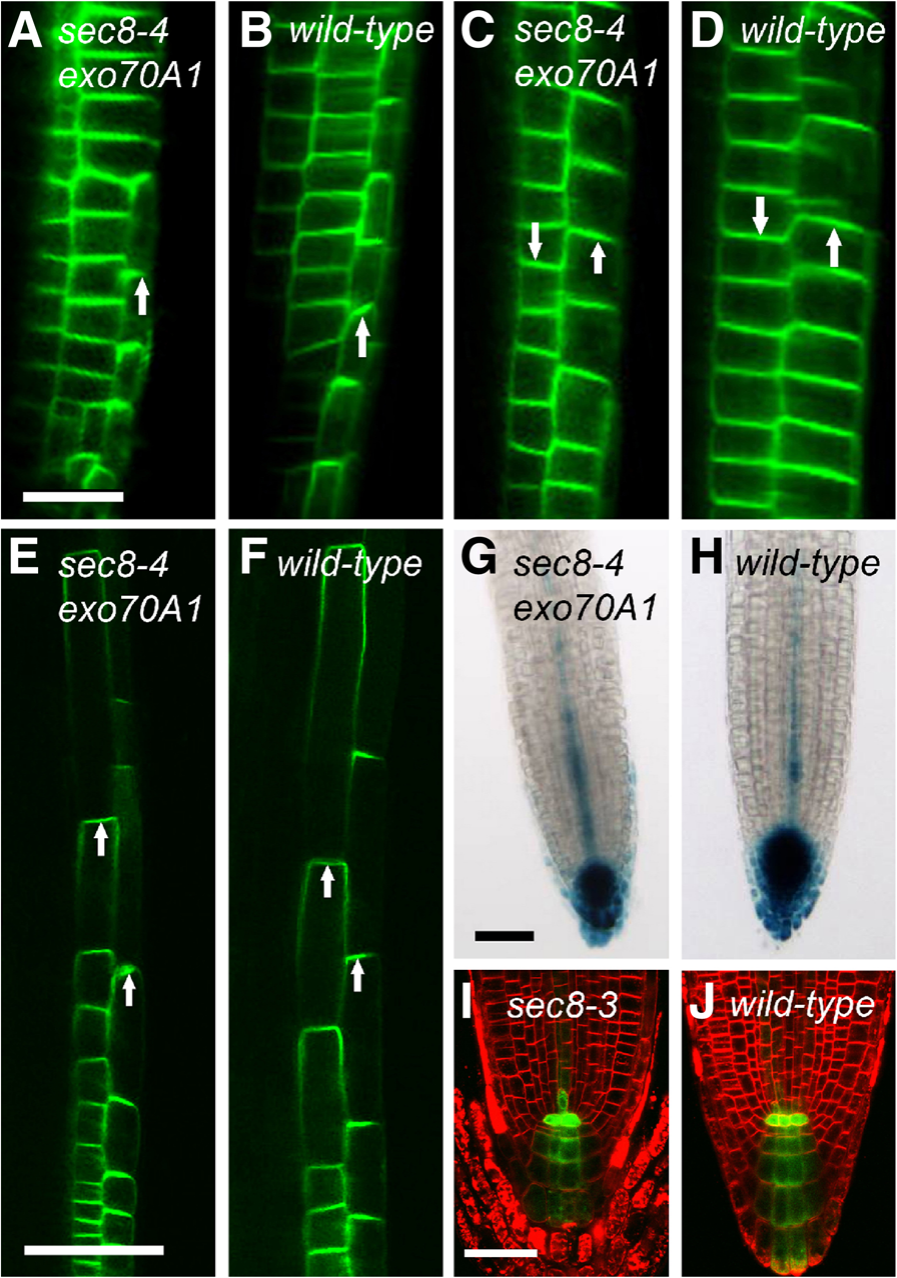
**Polarized localization of auxin transporters is evident in exocyst mutants. (A-F)** Polarized localization of *PIN2-GFP* (indicated by white arrows) in lateral root cap **(A, B)**, meristem **(C,D)**, and elongation zone **(E,F)** of *sec8-4 exo70A1* mutants **(A, C,** **and** **E)** is similar to that of wild-type siblings **(B, D,** **and** **F)**. In C-F, cortical (left) and epidermal (right) cell files are shown. Auxin response as indicated by *pDR5:GUS* **(G,H)** or *pDR5:GFP* **(I, J)** appears similar in *sec8-4 exo70A1* **(G)** and *sec8-3* **(I)**, compared to their wild-type siblings **(H, J)**. Bar for **A-D** = 20 microns; bars for **E&F, G&H**, and **I&J** = 50 microns. Localization of additional auxin transporters in exocyst mutant roots appear in Additional file 1: Figure S5, and Additional file 1: Figure S6. Additional *pDR5:GUS* images appear in Additional file 1: Figure S7.

We also examined the distribution and polar localization of several auxin transporters in exocyst mutants. The different auxin transport proteins (PINs, AUX1, and ABC transporters) achieve their individual and polarized localizations by delivery to the plasma membrane via functionally distinct secretory pathways [10,72-76], a subset of which could conceivably involve the exocyst. Consequently, we examined exocyst mutant lines containing labeled auxin transport proteins, PIN1-GFP, PIN2-GFP, PIN7-GFP, AUX1-YFP, and ABCG36-GFP to see if mislocalizations were evident. The polar localization within the cells and the pattern of distribution within the root tips (e.g., the MZ) of these auxin transport proteins in two exocyst mutant lines with severe root growth defects (*sec8-3*, and *sec8-4 exo70A1*) appeared similar to wild-type, as had previously been reported for the less severe *exo70A1* mutant [36] (Figure 7, Additional file 1: Figure S5 and Additional file 1: Figure S6). Notably, the expression of these auxin transporters in exocyst mutants was observed within smaller regions than wild-type, but consistent with an interpretation of a shortened wild-type distribution pattern, correlating with their shorter meristems.

Downstream of auxin transport and development of auxin gradients and auxin-mediated signaling is auxin-induced modulation of transcription, i.e. the auxin response. To further explore the possibility that the exocyst mutants have slower root growth because of a defect in auxin transport, we examined expression of *pDR5:GFP* and *pDR5:GUS*, reporters of auxin response. A subtly reduced region of auxin response peaking in the region of the QC, and indicated by *pDR5:GUS* expression was previously observed in *exo70A1* mutants [36]. In four exocyst mutant lines with the most severe root growth defects (*exo84b-1, sec8-1, sec8-3,* and *sec8-4 exo70A1*), we predicted a more profound alteration in the distribution/pattern of *DR5* expression if the exocyst mutant’s slower root growth was due to a defect in auxin response in the stem cell niche or MZ. However, when *pDR5:GFP* or *pDR5:GUS* expression was observed in the roots of these exocyst mutants, the reporters were observed in the same region of the root as wild-type with a peak of expression in the region of the quiescent center (Figure 7G-J, Additional file 1: Figure S7), albeit over a shorter length of the root tip, correlating with the smaller meristem. Any reduction in absolute expression level was not obviously correlated with the severity of the root growth defect. When auxin response was evaluated in seedlings grown in the dark (which reduces auxin production and acropetal auxin transport [77,78]), the significantly reduced level of *pDR5:GUS* expression (compared to that of light grown seedlings) and the pattern of that expression in the root tip were similar in *sec8-1* mutants and their wild-type siblings (Additional file 1: Figure S7). Thus, in both light and dark-grown seedlings, the root growth defect could not be correlated with an altered auxin response.

The maintenance and functioning of the stem cell niche is dependent upon auxin and vital to root growth. Within the niche, proximal stem cells next to the quiescent center contribute cells to the MZ, whereas distal stem cells produce cells that differentiate to become the columella cells of the root cap, identifiable by the accumulation of starch containing amyloplasts. Mutations that alter auxin levels or auxin transport to affect the root stem cell niche lead to aberrant starch localization, i.e. the presence of starch in the distal stem cells or the quiescent center, or conversely an increased number of non-differentiated (i.e. starchless) cell layers between the quiescent center and columella cells [42,78]. However, Lugol staining revealed starch localization in *exo70A1*, *sec8-3*, and *exo84b-1* mutants that was similar to that to Col-0 (n > 20 for each genotype; Additional file 1: Figure S8). This contrasts with the more diffuse presence of starch in root tips treated with NPA (Additional file 1: Figure S8B, C; [42,79]), in which the region of starch staining extends into the distal stem cells and lateral root cap. The exocyst mutants also did not demonstrate an increased number of cell layers that were undifferentiated between the quiescent center and the differentiated columella (Additional file 1: Figure S8G), unlike what has been observed in PIN and auxin biosynthesis mutants [42]. These results also distinguish exocyst mutants from the *icr1* mutant, in which altered auxin transport is associated with the disappearance of starch in the columella six days after germination [38]. Seven day-old exocyst mutants do have fewer layers of differentiated starch-stained columella cells compared to Col-0 (consistent with the overall observation of shorter developmental zones in the root meristem; Additional file 1: Figure S8-I), but unlike the *icr1* mutant the starch staining pattern persists in the mutants after two weeks of growth (data not shown). Thus, an auxin-mediated alteration in the stability and functioning of the root stem cell niche was not evident in exocyst mutants. This result is consistent with the root tip expression patterns of WOX5 and PLETHORA transcription factors (Figure 1G-L), which are believed to be regulated downstream of auxin signaling to control stem cell activity [42]. In summary, after assessing several independent characteristics linked to auxin, we found no evidence to support the hypothesis that the exocyst mutants’ shortened meristems have a basis in defective auxin transport or the failure to establish relative auxin maxima.

### The root growth defect in Exocyst mutants is sensitive to alterations in brassinosteroid signaling

One alternative to an auxin-related basis for reduced root growth rates in the exocyst mutants would be defective brassinosteroid signaling, which is closely linked to root cell elongation. Similar to exocyst mutants, both BR-deficient and BR-signaling mutants demonstrate reduced hypocotyl elongation and reduced apical hook formation (phenotypes associated with reduced cell elongation [27,80]), as well as altered meristem size and mature cell lengths in the root [47,48,81]. Furthermore, both the exocyst localization to the plasma membrane and brassinosteroid signaling are prominent in root epidermal cells [34,49], and the recycling of the brassinosteroid receptor, BRI1, at the plasma membrane is disturbed in exocyst mutants [36].

**Figure 8:**
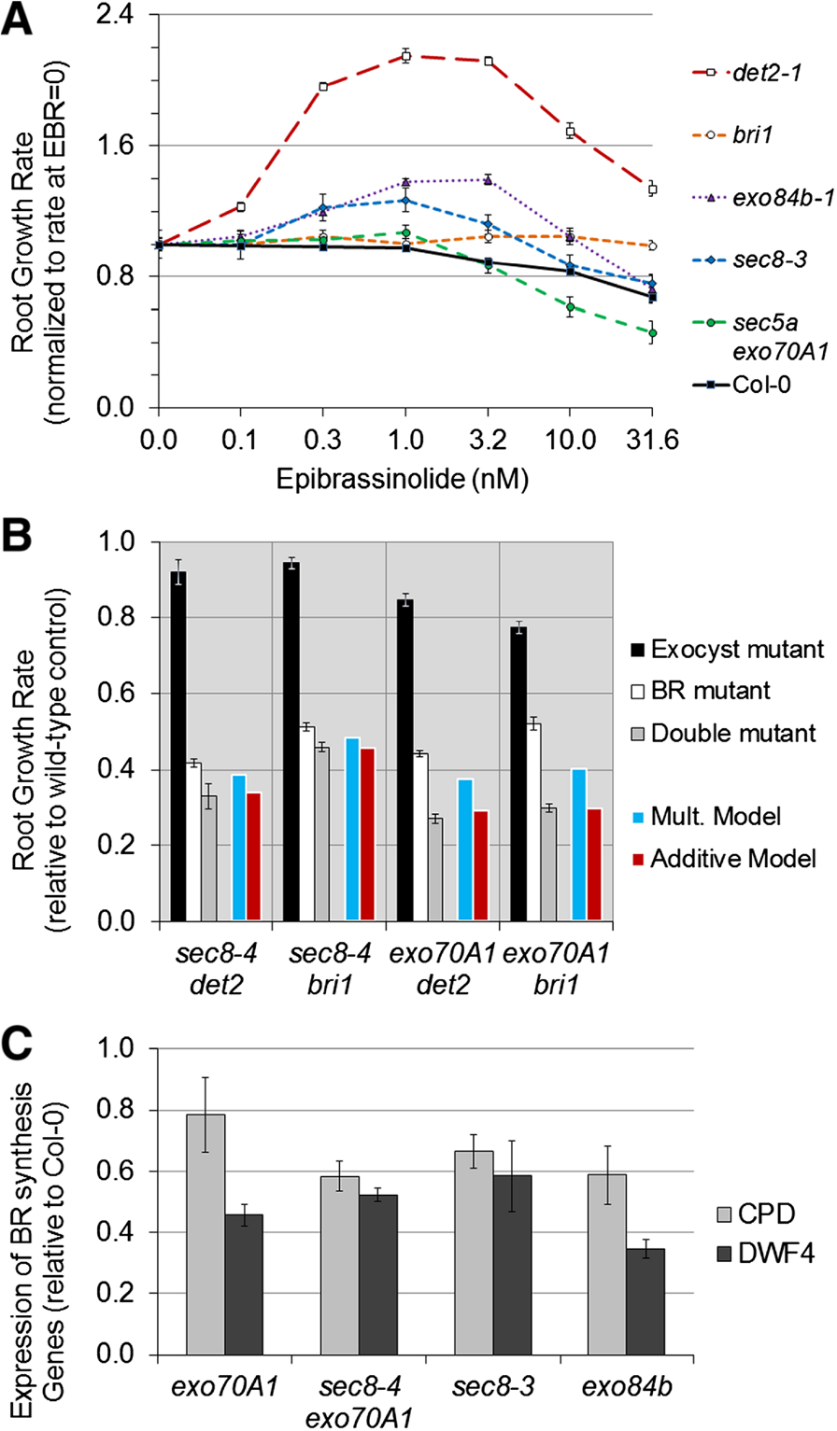
**Root growth in exocyst mutants shows a mild alteration in response to brassinosteroids. (A)** Dose–response of exocyst mutants to exogenous epi-brassinolide shows mild rescue. **(B)** Combinations of exocyst mutations (*sec8-4* or *exo70A1*) and brassinosteroid mutations (*det2-1* or *bri1*) lead to more severe root growth defects; the combinations with *exo70A1* are synergistic if interpreted by a multiplicative model. **(C)** qRT-PCR expression analysis indicates that the expression of brassinosteroid synthesis genes (*CPD* and *DWF4*) are reduced in exocyst mutants, contrary to what would be expected if the mutants had a defect in the canonical brassinosteroid signaling pathway.

A hypothesized role of the exocyst in brassinosteroid signaling would be supported by rescue of the root growth defect in exocyst mutants by exogenous hormone. And indeed, exocyst mutants with the most severe root growth defects, *sec8-3* and *exo84b-1*, demonstrated a mild rescue when grown on media containing low concentrations (1 or 3.16 nM) epi-brassinolide, whereas wild-type Col-0 plants showed a decreased growth rate in this same concentration range, and the brassinosteroid receptor mutant, *bri1*, showed no response (insensitivity) (Figure 8A). However, the rescue was small in absolute terms (Additional file 1: Figure S9), with the partially rescued growth rate remaining less than wild-type, and far less than the rescue observed for the *det2-1* brassinosteroid synthesis mutant. In relative terms, exogenous brassinosteroid stimulated a significant (p < 0.05, *t*-test, n = 20-44) increase in growth rate of 27 and 39 percent over the untreated controls in *sec8-3* and *exo84b-1,* respectively, providing at least some support for the hypothesis.

We next overexpressed the kinase BSK3 in *sec8-3* and *exo84b-1* mutants to determine whether the observed rescue was linked to the canonical BRI1 signaling pathway. BSK3 is phosphorylated by the BRI1 receptor to activate downstream BR-induced transcription, and its overexpression rescues both BR biosynthetic (*det2*) and BR receptor (*bri1-5*) mutants [82]. In both the *sec8-3* and *exo84b-1* mutants, the over-expression of *BSK3* resulted in only very slight (14 percent and 9 percent, respectively) but significant (p < 0.01, *t*-test, n = 41-147) increases in root growth rate (Additional file 1: Figure S10). In contrast, there was no significant difference in the growth rates of the wild-type siblings when seedlings with or without the *BSK3* construct were compared. Thus, consistent with the results for treatment with exogenous epi-brassinolide, *BSK3* overexpression provided a slight rescue of the exocyst mutant phenotype.

We also tested for a genetic interaction between brassinosteroid signaling and the exocyst. A quantitative synergistic genetic interaction is identified when a double mutant has a more extreme phenotype than would be predicted by a neutrality function, a function that predicts the phenotype when the two mutations do not interact [83]. Neutrality functions based upon multiplicative or additive models have been used to study genetic interactions [84,85], with the multiplicative model identified as being more appropriate for predicting functional relationships [83]. Seedlings possessing a combination of an exocyst mutation (*exo70A1-1* or *sec8-4*, each associated with a mild root growth defect) and a brassinosteroid-related mutation (*det2-1*, affecting BR synthesis, or *bri1,* affecting BR signaling) were evaluated to determine if the two mutations had a synergistic effect on reducing root growth. In each of the four combinations, the double mutant demonstrated a more severe root growth defect than either of the single mutants (Figure 8B, p < 0.01, *t*-test, n = 20-75). The growth rates for the double mutants were not significantly different from what would be predicted by an additive model (p > 0.10, z-test, for all four double mutant combinations), nor were the growth rates for *sec8-4 det2* and *sec8-4 bri1* mutants statistically different from prediction by multiplicative model prediction (p > 0.05, z-test). However, the root growth defect was significantly more severe than predicted by the multiplicative model for *exo70A1 det2* and *exo70A1 bri1* mutants (p < 0.001, z-test). Thus, any synergistic interaction is not very robust, as it is only recognizable under the multiplicative model and only in combination with the *exo70A1* mutant, suggesting that any functional interactions between exocyst-mediated events and brassinosteroid signaling to influence root growth are limited, and perhaps indirect.

The biosynthesis of brassinosteroids is known to be under feedback control, such that decreased signaling via BRI1, BSK3, and downstream transcription factors leads to the increased synthesis of BR biosynthesis genes [86,87]. If the exocyst mutations are causing a defect in either brassinosteroid availability to receptors, perception, or downstream signaling through this pathway, then one would predict that the exocyst mutants should demonstrate an elevated expression of BR biosynthesis genes. To test this prediction, qRT-PCR was performed on cDNA from mutant roots to evaluate the expression of DWF4 and CPD, two brassinosteroid biosynthesis genes. Rather than being elevated, expression of these genes was found to be slightly depressed in the exocyst mutants (Figure 8C), indicating that BR signaling was not reduced globally in the root, although this does not rule out the possibility that signaling is reduced in some regions or cell types of the root.

To better assess whether the root growth defects in exocyst mutants phenocopy those associated with defects in brassinosteroid signaling, cell lengths along cortical cell files obtained by confocal microscopy were evaluated for *det2-1* and *bri1-2* mutants and compared to those of exocyst mutants (Figure 1B, Figure 2). This revealed that, at the cellular level, the root growth defect in the brassinosteroid mutants is distinct from that in exocyst mutants. The shorter root meristem in brassinosteroid mutants is primarily due to shortened cell length, whereas the shorter meristem in exocyst mutants is due to fewer cells. A decreased cell production rate in brassinosteroid mutant roots is associated with a prolonged cell cycle (consistent with previous reports: [47,48]) that is not seen in exocyst mutants. The mature cortical cells of brassinosteroid mutants had length-to-width ratios that were significantly greater than Col-0 or any of the exocyst mutants (Figure 2L, *t* test, p < 0.001, n = 7). On the other hand, both the exocyst and brassinosteroid mutants demonstrate a reduced mature cortical cell length compared to wild-type (Figure 2E, p < 0.001, *t*-test, n = 7) and this reduction is partially due to slower elongation in both cases (Additional file 2).

To determine if the partial rescue of root growth rate with exogenous BR could be the result of an effect solely on exocyst-dependent cell expansion in the EZ, roots treated with and without 1 nM epibrassinolide were studied. Root growth rates were ascertained and mature cortical cell lengths were measured. The growth rates of *exo84b-1*, *sec8-4 exo70A1*, and *sec8-3* were 35-49% higher in the epi-brassinolide treated groups, compared to the untreated groups (n = 12-18 roots for each genotype/treatment group), but the corresponding mature cortical cell lengths were only 12-19% longer in the exocyst mutants treated with epi-brassinolide, compared to untreated controls. Thus an effect on mature cell length could only account for about one-third of the observed rescue, suggesting the added epi-brassinolide did not act exclusively to increase cell elongation, but also increased the cell production rate.

An alternative target for linking the exocyst to BR signaling is *BREVIS RADIX (BRX)*, a plasma membrane-associated protein that is subject to endocytic recycling [88,89], and upon auxin treatment translocates to the nucleus [86] where it potentially mediates cross-talk between auxin and brassinosteroid signaling pathways [90]. Mutant *brx* seedlings have slower growing roots with shorter meristems and shorter mature cell lengths [91]. Consequently, we tested whether the exocyst might be involved in the cycling of BRX to the plasma membrane to affect root growth by attempting to rescue the root growth defect in exocyst mutants using methods that have been shown to rescue the *brx* mutant: over-expressing BRX; introducing a recessive null LONG HYPOCOTYL 5 (*hy5*) mutation [88]; and growing the mutants on a medium with a more basic pH [92]. In each case, rescue of the exocyst mutant phenotype was not achieved, indicating that the root growth defect in exocyst mutants is not related to reduced BRX activity (Additional file 1: Figure S11).

## Discussion

### The exocyst’s role in primary root growth occurs within two developmental contexts

The developmental progression of root cells behind the root cap can be visualized sequentially along cell files beginning at the quiescent center: division in the meristem proper, slow constant elongation in the transition zone, exponential elongation in the elongation zone, followed by cessation of expansion and cell maturation. As revealed by an examination of the roots of exocyst mutants, the exocyst has two distinctive roles in root growth that occur in two different stages of root development. First, the exocyst is involved in determining the root cell production rate by helping to establish the size of the root meristem, and therefore the number of cells that are dividing. This is distinct from an effect on the rate of cell division, which is not reduced in exocyst mutants (i.e. the cell cycle length is not prolonged in the mutants). Second, the exocyst is involved in determining mature cell length, primarily by enabling an exponential rate of cell elongation in the elongation zone (EZ).

**Figure 9:**
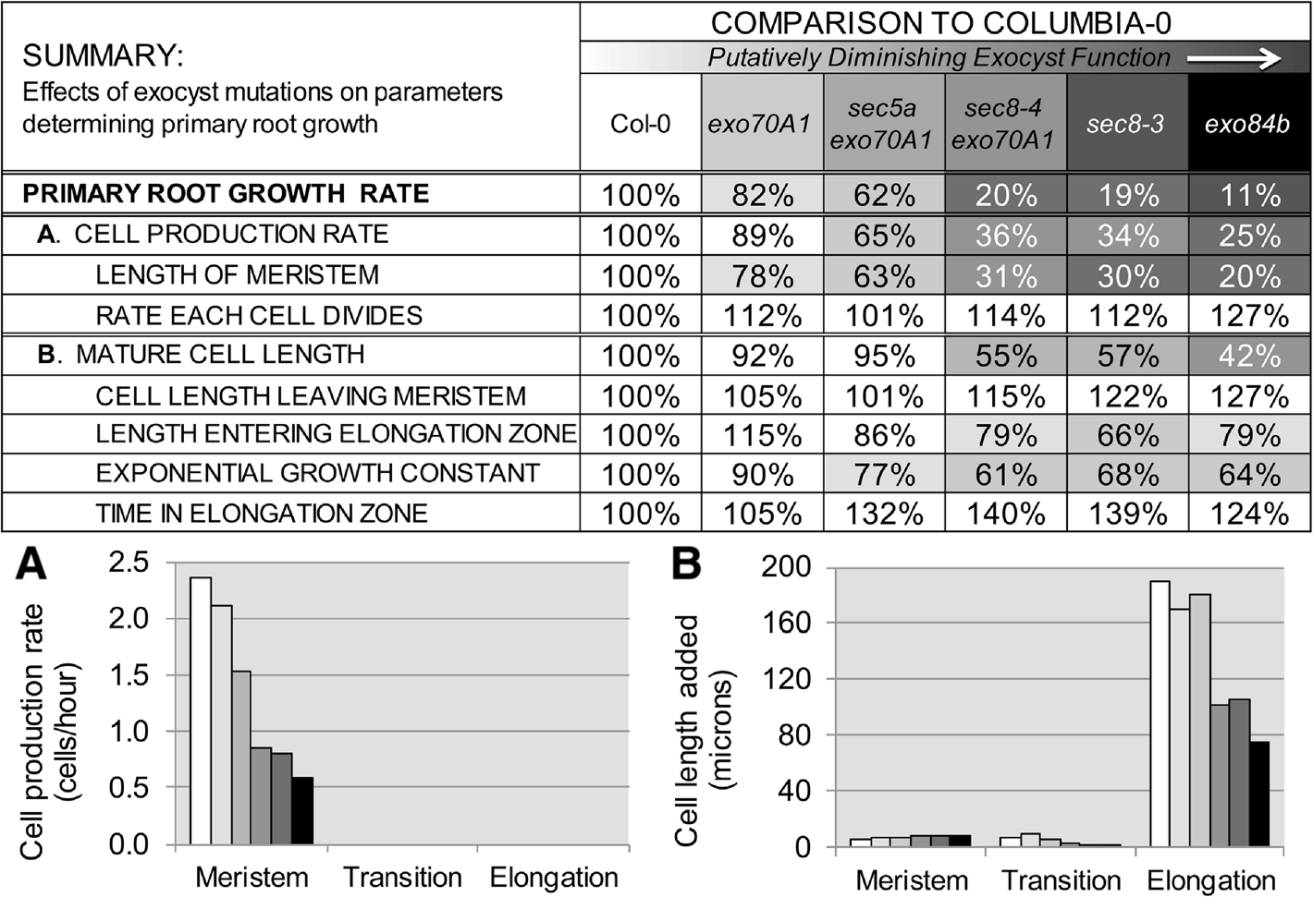
**The exocyst’s role in root growth.** The exocyst is required for root growth in two developmental contexts to affect **(A)** cell production; and **(B)** cell length. Table above graphs shows how parameters determining primary root growth are affected by exocyst mutations; values shown are percentages of the Col-0 value for each parameter. Bars in graphs **A** and **B** represent (from white to black): Col0 (wild-type), *exo70A1*, *sec5a exo70A1, sec8-4 exo70A1, sec8-3* and *exo84b-1*. In the meristem exocyst mutations reduce the cell production rate **(A)**, but not the length of cells in the cortical cell file **(B)**. However, in the transition and elongation zones, where new cells are rarely produced in either wild-type or exocyst mutant roots, mutation of the exocyst leads to severely reduced cortical cell elongation **(B)**.

There is suggestive evidence that the specific processes involving the exocyst in these two developmental events (i.e. meristem size determination and cell expansion in the EZ) are not the same. Notably, the series of exocyst mutants differentially affect the two processes. Meristem size is reduced in parallel with the reduction in root growth rate over the entire range of exocyst mutants evaluated. In contrast, rate of cell elongation is reduced in parallel with growth rate only for the exocyst mutants with less severe root growth defects; whereas the three exocyst mutant lines with the most severe defects in root growth (*sec8-3*, *sec8-4 exo70a1*, and *exo84b*) share the same dramatically reduced cell elongation rate. In other words, there is a mismatch in severity of the two developmental defects across the range of mutants evaluated (Figure 9), with the reduction in meristem size appearing to be a continually graded response, and cell elongation influenced by an apparent threshold of exocyst activity.

We considered two ways the exocyst might conceivably influence both developmental events through the same process. First, the exocyst’s role in helping determine the size of the growth zones might result in exocyst mutants not only having a reduced cell production rate because of a shortened meristem, but also a reduced mature cell length resulting from a shortened elongation zone (allowing less time for root cells to elongate). However, such an explanation is not supported by our data: cortical cells in the mutants spend a longer period in the elongation zone, but elongate at a slower rate. Second, the exocyst could affect cells in both the elongation zone and the meristem as a general facilitator of cell expansion. Such a role could conceivably slow the cell cycle and the cell production rate in the meristem by slowing cytokinesis or entry into mitosis based on cell size. However, again this is not the case: neither cell elongation rates nor the cell cycle is slower in the meristems of exocyst mutants. This argues against a general role for the exocyst in cell expansion, but rather is consistent with an interpretation in which the exocyst is required for the remarkable cell expansion rates specifically associated with the EZ. Together, these results favor the hypothesis that the exocyst functions differentially to affect the size of the root growth zones and, via a different process, the rate of cell elongation in the elongation zone. Given the exocyst’s likely role in facilitating vesicle trafficking to the plasma membrane, one potential explanation for such differential effects would be based on the presence of developmental stage-specific cargoes that are dependent on the exocyst for transport and subsequent activity. In such a scenario, the pleiotropic, multi-functional nature of the exocyst would be due to its usage by the organism to correctly traffic different sets of developmentally-appropriate vesicle cargoes.

### The exocyst affects the size of the root growth zones by a mechanism that could not be directly linked to auxin

Plant hormones play dominant and interacting roles in regulating the size of the root meristem and other root growth zones by controlling and coordinating specific processes (reviewed in [3-5,7]). For example, auxin acts on the stem cell niche [42], promotes cell division in the meristem, and targets the elongating epidermal cells in the root transition/elongation zone during a gravitropic response [68]; cytokinins act at the transition zone of the stele [62]; and brassinosteroid perception in the epidermis mediates its effect on meristem size [48]. Among the many hormone-mediated control pathways that affect root growth, auxin and brassinosteroid signaling were considered the most likely candidates for involvement of the exocyst. Plasma membrane localization of exocyst subunits (EXO70, SEC6, SEC8, EXO84) is prominent in the root epidermis [34], coincident with important sites for auxin and brassinosteroid signaling. Additionally, in the root, the exocyst has been linked to acropetal auxin transport, as well as to recycling of the PIN1 and PIN2 auxin efflux carriers and the BRI1 brassinosteroid receptor to the plasma membrane [36]. Consequently, we hypothesized that the root growth defect in exocyst mutants might be largely the result of a defect in PIN-mediated auxin transport and signaling, or possibly altered brassinosteroid signaling.

We assessed exocyst mutant roots for indicators of altered auxin signaling, including: (1) altered sensitivity to either exogenous auxins, an auxin transport inhibitor, cytokinin, or ACC; (2) altered localization of auxin transporters, the pWOX5::GFP marker for quiescent center identity, auxin-regulated PLT transcription factors, or DR5-driven markers of auxin response, (3) rescue with a *hy5* mutation, which is associated with elevated auxin-responsive transcription, and (4) the aberrant presence of starch in the columella initials. Exocyst mutants treated with exogenous hormones had dose–response curves that were similar to wild-type. Localization of markers for auxin responsiveness; PIN, AUX1, and ABCG36 transporters; quiescent center identity; and PLT expression occurred over shorter root regions coincident with the shorter root growth zones, but, otherwise, the localization patterns were not remarkably different in exocyst mutants compared to wild-type. The *hy5* mutation failed to exert any detectable rescue of the exocyst mutant phenotype. Increased staining of starch in the columella initials, which is indicative of altered auxin signaling in the vicinity of the stem cell niche, was not observed in the exocyst mutants. Although we did not conduct experiments addressing a possible link between the exocyst and ABCB auxin transporters, mutation of ABCB transporters leads to phenotypes not consistent with our observations of exocyst mutants (e.g., longer root hairs [91,93], enhanced gravitropism [94,95], sporadic root curvature [94], prominent reduction of the number and growth of lateral roots [94,96,97]). It must be acknowledged that direct assessment of auxin signaling by the TIR complex was not addressed in the range of experiments performed on exocyst mutants. However, the severe root growth defect observed in exocyst mutants could not be convincingly associated with any of several indirect indicators of altered auxin transport or signaling.

Drdová, et al. [36] demonstrated that the recycling of PIN1 and PIN2 proteins to the plasma membrane from brefeldin-A compartments is delayed in both *exo70A1* and *sec8-1* mutants. Our results do not necessarily contradict these data, but do argue that a role for exocyst activity in PIN trafficking is not a major driver influencing root growth rate. The lack of a clear connection to altered auxin signaling in exocyst mutants also contrasts with the mutant phenotypes of the Rop GTPase- and SEC3-interacting scaffold protein ICR1 [37], which dramatically perturbs root meristem pattern (e.g., disorganized *WOX5* and *DR5::GUS* expression) and auxin transport (e.g., altered PIN1 and PIN2 subcellular distribution) [38]. Thus, our observations also argue against the exocyst as the primary link between ICR1 and auxin. However, auxin regulates a broad range of developmental and physiological processes, and auxin transport and signaling is correspondingly complex [98,99]. Thus, we cannot rule out altered auxin transport or signaling in exocyst mutants as a contributor to their root growth defect; but we conclude that the exocyst is unlikely to be directly linked to root growth via a currently understood process in auxin transport and signaling.

### Enhanced brassinosteroid signaling can partially compensate for, but not fully rescue, the root growth defect in exocyst mutants

We also investigated the possibility that the exocyst is involved in the transport of brassinosteroids to the plasma membrane where they bind BRI1-type receptors [13], or in the plasma membrane placement of those receptors, to affect brassinosteroid signaling [81]. Consistent with this possibility, exocyst mutants with severe root growth defects did demonstrate a small, but statistically-significant, rescue of the mutant phenotype with the application of exogenous epi-brassinolide, and an altered dose–response curve. Overexpression of the BR signaling kinase, BSK3, to activate BR signaling downstream of BRI1 receptor also caused a slight increase in root growth of exocyst mutants that was not seen in their wild-type siblings. Evaluation of double exocyst-brassinosteroid mutants for a genetic interaction provided a somewhat ambiguous result: the double mutants, *exo70A1 det2-1* and *exo70A1 bri1*, had growth defects that were more severe than would be predicted from the growth rates of single mutants by a multiplicative model, but not by an additive model. These data support a functional interaction between the exocyst and brassinosteroid signaling, but are consistent with the possibility that this interaction is indirect.

Three lines of evidence suggest the exocyst does not directly interact with brassinosteroid signaling to affect root growth. First, qRT-PCR analysis indicates that the expression of brassinosteroid synthesis genes is not elevated in exocyst mutant roots. This is contrary to the expectation for defects in brassinosteroid signaling, because feedback control dictates increased expression of the BR synthesis genes when the BR signal is attenuated (86). Second, although the dynamics of BRI1 cycling is altered from wild-type in exocyst mutants treated with BFA [36], the ultimate localization of BRI1-GFP to the plasma membrane is not significantly altered in exocyst mutants with the most severe root growth defects (Additional file 1: Figure S9). Third, a detailed comparison of the cortical cell length profiles reveals important differences between exocyst and brassinosteroid mutants. Shortened meristems in exocyst mutants are due to fewer cells (not an altered cell cycle), whereas shortened meristems in brassinosteroid mutants (*det2-1* and *bri1-2*) are primarily due to shortened cell lengths (and are also associated with a prolonged cell cycle). Both exocyst and brassinosteroid mutants demonstrate a reduced mature cortical cell length and a slower rate of cell elongation in the elongation zone, but the rate of cell elongation is much more dramatically reduced in exocyst mutants, and the final cell length-to-width ratio is also distinct. A specific role for the exocyst in trafficking of the upstream regulatory protein, BRX, was discounted when several experimental manipulations known to rescue the *brx* mutant failed to rescue the exocyst mutant root growth phenotype. Together these observations argue that the exocyst root growth phenotype is not primarily driven by defects in brassinosteroid signaling.

The partial rescue of the growth rate defect by application of low concentrations of epi-brassinolide deserves further consideration. Recent evidence suggests that in addition to the canonical intracellular BR signaling via a kinase cascade [9,81], there is also a non-cell autonomous BR-induced signal from the root epidermis to the steele [48], a BR-induced fast response involving activation of a plasma membrane P-ATPase [100], and a BR induced cyclic GMP-mediated Ca^2+^ signaling cascade [101]. A direct role for the exocyst in any of these pathways could explain the observation that epi-brassinolide treatment provides a stronger rescue than the specific induction of the kinase cascade via BSK3 overexpression. Alternatively, rescue of the exocyst phenotype could be accomplished indirectly, by BR induction of a process that can partially compensate for loss of exocyst function. As an example, in *S. cerevisiae*, overexpression of SRO7 (an Lgl family protein that interacts with t-SNAREs) can suppress the growth defects of multiple exocyst mutants [102]. Induction of a similar gene function in Arabidopsis by BR would have a larger effect if exocyst activity is reduced, explaining both the partial epi-BR and BSK3 rescue.

### Potential alternative roles for the exocyst in root growth

Overall, despite a role for the exocyst in PIN and BRI1 trafficking in root epidermal cells (36), the root growth defect in exocyst mutants does not appear to be explained by a simple inhibition of the known auxin/cytokinin- and brassinosteroid-based mechanisms that help define the developmental activities of the root meristem and elongation zone [40,48]. However, observation of the constellation of root growth characteristics seen in exocyst mutants, occurring with an apparent independence from these phytohormones pathways, is not unprecedented. The exocyst mutant phenotype is mimicked in seedlings overexpressing the UPBEAT transcription factor [103]. Overexpressing UPBEAT down-regulates expression of class III peroxidases in the root’s elongation zone, leading to an altered balance of reactive oxygen species (ROS), a resultant shift in the root’s transition zone (i.e. between proliferation and differentiation) and reduced root growth. UPBEAT is hypothesized to act both directly (via expression of peroxidases secreted to the apoplast) and indirectly (via ROS signaling) to modify cell walls. The possibility of exocyst involvement in a ROS-mediated mechanism to affect root growth, for example, a role in the secretion of peroxidases in the root’s elongation zone, deserves exploration.

The exocyst mutant root phenotype (including reduction in size of growth zones and reduced rate of cell elongation, occurring without prolonged cell cycle duration, altered stem cell niche, or a prominent defect in auxin transport) is also strikingly similar to that reported for seedlings stressed by growth on medium containing elevated concentrations of ammonium [104]. There is no obvious link between the exocyst and ammonium metabolism, but there could be a connection between the exocyst and a plant’s response to abiotic stress. Repression of root cell elongation occurs in response to a variety of environmental stresses [104-106], and setting the size of the growth zones is considered to be the key regulatory act for root growth acclimation to environmental conditions [107]. Thus, the primary characteristics of root growth that are affected upon inhibition of exocyst function are coincident with those root growth characteristics that are adjusted in response to environmental stress. The potential involvement of the exocyst in the root’s growth response to abiotic environmental stress is an interesting possibility.

Environmental stressors elicit a host of varied signaling pathways, involving hormone-modulated systems [2,108,109], cell-type developmentally-specific transcriptional modules [1,103], and ROS [110,111]. However, similarities in the root growth response suggest these pathways may ultimately converge to a common set of downstream mechanisms that alter growth. Central to the downstream mechanisms, particularly in the root’s transition and elongation zones, are secretory processes that deliver material to form the cell wall matrix (e.g., secreted pectins and hemi-celluloses), and/or proteins that modify the matrix to promote cell wall loosening and expansion (e.g. expansins, xyloglucan endotransglycolase/hydrolases, endo-(1,4)-β-D-glucanases, and peroxidases) [14,112-117]. Modulating the activity of the exocyst to affect these downstream secretory events and cell wall expansion could be integral to the mechanisms that ultimately allow for growth that is both developmentally coordinated and environmentally responsive. The framework for exocyst function in the growing root established here will help better define future work to address this and the aforementioned possibilities.

## Conclusions

A complex network of interacting, overlapping, feedback-controlled, and often hormone-mediated mechanisms have evolved to control and maintain primary root growth [3-5,118]. In the midst of this complex system, perhaps at multiple sites, lies the exocyst, a putative molecular tether that facilitates secretory vesicle delivery for fusion to the plasma membrane in both plant [19,34,35] and non-plant species [16-18,23]. Mutate a component of the exocyst and a dramatic reduction in root growth rate results, in certain cases down to a mere 11% of the growth rate observed in wild-type roots. At the same time, the resilience of the system is revealed in exocyst mutants: they maintain the overall meristematic, growth zone, and tissue layer structure of wild-type roots, although the number of cells in these regions is reduced in the mutants. Evaluation of cortical cell files reveals that the slower growth rates in exocyst mutants arise because their meristematic cell production rates are lower, and their mature cells are shorter compared to wild-type roots. This analysis provides evidence that the exocyst functions in two different developmental contexts to affect root growth, seemingly independent of auxin and brassinosteroids, influencing both the size of the growth zones and the rate of cell elongation.

## Methods

### Plant material and growth conditions

Lines of Columbia-0 ecotype of *Arabidopsis thaliana* with T-DNA insertions and other mutants were obtained from the SALK Institute [119]: *exo70A1-2* (At5g03540) SALK 135462; *sec5a-1* (At1g76850) SALK 010127; *sec8-1* (At3g10380) SALK 057409; *sec8-3* (At3g10380) SALK 026204; *sec8-4* (At3g10380) SALK 118129; *sec8-6* (At3g10380) SALK 091118; *bri1* (At4g39400) SALK 003371; *det2-1* (At2g38050) CS6159; *pgm-1* (At5g51820) CS210; *arg-1* (At3g68370) SALK 024542C. The exo84b-1 line was a GABI-Kat line [120], provided from the Zarsky Lab (30). Exocyst T-DNA insertion sites for exocyst related SALK lines were verified by sequencing. PCR genotyping for exocyst mutant alleles was performed as previously described [27,28]. Homozygous exocyst mutants with severe root growth defects (i.e. *sec8-1, sec8-3, exo84b-1*) are virtually sterile and were obtained in the progeny of self-crossed heterozygous plants. The homozygotes could be readily identified by root growth and root hair phenotypes and were consistently verified by spot-checking with PCR). Mutant lines *aux1-7* and *pin2-1* were provided by M. Ivanchencko. Marker lines pPLT1:gPLT1-YFP, pPLT2:PLT2-YFP and pWOX5:GFP were provided by Y. Du and B. Scheres; pPIN7:PIN7-GFP was provided by Wendy Peer, Purdue University; PIN1-GFP, PIN2-GFP and AUX1-YFP were provided by S. Robert and N. Raikhel; ABCG36/PEN3-GFP was provided by B. Underwood and S. Somerville; pDR5:GFP and pDR5:GUS were from M. Ivanchenko. Marker BRI1-GFP was provided by J. Chory; p35S:BSK3-YFP was provided by Z. Wang; and p35S:BRX-GFP was provided by A. Amiquet.

Arabidopsis seeds were surface-sterilized, stratified at 4°C for 3–5 days, and planted on growth media (1x MS, 2% (w/v) sucrose, and vitamins) or soil as previously described [28]. Serial dilutions of hormones were prepared and added to media cooled to 50°C prior to pouring plates. Phytohormones: 3-indoleacetic acid (IAA), naphalene acetic acid (NAA), N-6-benzyladenine, 24-epibrassinolide, 1-aminocyclopropanecarboxylic acid (ACC), and naphthylphthalamic acid (NPA), as well as brefeldin A (BFA) were all obtained from Sigma-Aldrich. Plants were grown in a climate chamber at 22°C under long-day conditions (16 hr. of light per day).

### Microscopy

Root growth rates at 7 days of growth were calculated from the differences in root lengths observed on days 6 and 8, and captured with a Canon Power Shot A710IS digital camera, or with a Moticam 1000 camera attached to a Zeiss Stemi SV 11 dissecting microscope. For the analysis of root tips stained for GUS activity or starch, seedlings were fixed in 0.3% formaldehyde in 0.33 M phosphate buffer pH 7.2 for 30 min at room temperature, and then rinsed three times in 50 mM phosphate buffer pH 7.2. GUS activity was analyzed after staining the fixed seedlings overnight at 37°C and then cleared as described by Malamy and Benfey [121] and modified by Ivanchenko, et al. [122]. Starch was stained by placing fixed root tips in Lugol stain [0.34% (w/v) I_2_ with 0.68% (w/v) KI in H_2_O] for 15 minutes. Light microscopy of root tips stained for GUS activity or starch was performed on a Zeiss Axiovert microscope with differential interference contrast optics. Live roots stained with propidium iodide (10 μg/ml for at least 15 minutes) or containing fluorescent markers were imaged at the Confocal Microscopy Facility of the Center for Genome Research and Biocomputing and the Environmental and Health Sciences Center at Oregon State University using a Zeiss LSM510 META with Axiovert 200 motorized microscope with version 3.2 LSM software (National Institute of Health grant number 1S10RR107903-01). Root lengths or cell dimensions in digital images were measured using ImagePro 6.2 software (MediaCybernetics, http://www.mediacy.com).

### Cortical cell file analysis

Four distinguishable developmental zones - the meristem, and the transition, elongation, and maturation zones [5,41] - were identified by examining cortical cell lengths along a cell file. Analyzed from the tip, cells lengths observed within a two-fold range were considered to be dividing and were identified as meristematic cells. Following the meristem, a transition zone (some have called the distal elongation zone) was identified as the region where cell lengths no longer appeared to divide to produce two smaller cells, but instead showed a slow steady increase in length as one proceeded up the cell file. The transition zone ended and the beginning of an accelerated elongation zone was identified by a series of cells that increased exponentially in length. This elongation zone ended and the maturation zone began when there was no longer an exponential increase in cell size, but instead cell lengths varied around an average mature cell length, whereas in neighboring cell layers root hairs or lateral root formation were often observed. Analysis of growth parameters from cortical cell length profiles is described in detail in Additional file 2. Briefly, the cell production rate was calculated by dividing the root growth rate by the average mature cell length [123,124]. An estimate of the length of the cell cycle was obtained by dividing the cell production rate by the number of dividing cells (i.e. the number of cells in the meristem) and multiplying by the ln(2) [46]. The average time interval between each cortical cell leaving the meristem to enter the transition and elongation zones was estimated as the inverse of the cell production rate [123,125,126]. Linear regression analysis of the cell length data after logarithmic transformation then provided a basis for determining the exponential rate constant for elongation in the elongation zone, and a means for comparison of the elongation curves between genotypes.

### qRT-PCR

Whole roots were harvested from 8 day old seedlings growing on MS plus 2% sucrose medium on vertically oriented plates, and immediately frozen in liquid nitrogen, and stored in −80 degree C freezer. Three biological replicates (50–100 mg tissue each = up to ~100 roots) were collected for each genotype, with each biological replicate composed of the pooled root samples from a particular planting of seeds from a unique parent plant. RNA was collected from the frozen tissue by grinding it under liquid nitrogen using mortar and pestle, adding TRIzol reagent (Invitrogen Cat No. 15596–018), and collecting the aqueous phase of a chloroform extraction. The RNA was precipitated from the aqueous phase with the addition of isopropyl alcohol, pelleted by centrifugation, washed with 75% ethanol, and briefly dried. Each pellet was redissolved in RNase-free water, and purified with QIAGEN RNeasy MinElute Cleanup Kit, Cat No. 74204 following manufacturer’s instructions. Quality and quantity of the purified RNA was verified using an Agilent Bioanalyzer 2100 (RIN’s: 9.8-10.0). RNA was treated with DNaseI (Invitrogen, Cat No 18068–015) prior to synthesis of cDNA using the Superscript III First-Strand Synthesis System (Invitrogen, Cat No. 18080–051) per manufacturers protocol.

The qRT-PCR was performed using the BioRad CFX96 cycler. Primers (listed in Additional file 3) were designed to amplify selected sequences using the following criteria: Tm of 59–61 C, primer lengths of 20–25 nucleotides, guanine-cyctosine contents of 40-55%, amplifying near the 3′ end of the transcripts to produce an PCR amplicon length of 55–150 bp, and covering an exon-exon junction if possible. Quantification cycle values from qPCR were obtained for three technical replicates for each of the 3 biological replicates for each gene evaluated per plant genotype. Efficiency for each qPCR reaction was determined by evaluation of a dilution series run on the same plate as the samples (Efficiencies are included in Additional file 3). *TIP41* (At4g34270), *SAND* (At2g28390), and a protein coding gene (At4g33380) were selected as reference genes based upon their documented stability of expression and successful use in qRT-PCR studies of roots [127-131]. Cq values were converted to relative quantities by comparing Cq values from each exocyst mutant line to the corresponding Cq value for Col 0. Calculation of these relative quantities took into account qPCR efficiencies, utilizing the qBase framework [132].

## Additional files

Additional file 1:
**Supplemental Figures. Figure S1** - Exocyst mutant seedling images; **Figure S2** - Plethora-YFP in mutant root tips; **Figure S3** - T-test comparisons of root growth characteristics; **Figure S4** - Phytohormone dose-response curves of exocyst mutants; **Figure S5** - Fluorescently labeled auxin transporters in *sec8-4 exo70A1* root tips; **Figure S6** - Fluorescently labeled auxin transporters in *sec8-3* root tips; **Figure S7** - pDR5-GUS in dark and light grown seedling root tips; **Figure S8** - Lugol-stained starch in root tips of exocyst mutants; **Figure S9** - BRI1-GFP in mutant root tips and epibrassinolide dose-response; **Figure S10** - Effect of BSK3 overexpression on root growth in exocyst mutants; **Figure S11** - Effect of BFA on root growth in exocyst mutants. Additional file 2:
**Using cortical cell length profiles to compare root growth characteristics.**
Additional file 3:
**qRT-PCR primers.**

## Abbreviations

MZ: Meristematic zone
TZ: Transition zone
EZ: Elongation zone
ABFA: Brefeldin

## References

[1] Dinneny J, Long T, Wang J, Jung J, Mace D, Pointer S, Barron C, Brady S, Schiefelbein J, Benfey P (2008). Cell identity mediates the response of Arabidopsis roots to abiotic stress. Science.

[2] Wolters H, Jürgens G (2009). Survival of the flexible: hormonal growth control and adaptation in plant development. Nat Rev Genet.

[3] Garay-Arroyo A, Sánchez M, García-Ponce B, Azepeitia E, Alvarez-Buylla E (2012). Hormone symphony during root growth and development. Dev Dyn.

[4] Perilli S, Di Mambro R, Sabatini S (2012). Growth and development of the root apical meristem. Curr Opin Plant Biol.

[5] Petricka J, Winter C, Benfey P (2012). Control of Arabidopsis root development. Annu Rev Plant Biol.

[6] Rymen B, Sugimoto K (2012). Tuning growth to the environmental demands. Curr Opin Plant Biol.

[7] Ubeda-Tomás S, Bennett M (2010). Plant development: size matters, and it’s all down to hormones. Curr Biol.

[8] Worden N, Park E, Drakakaki G (2012). Trans-Golgi network – an intersection of trafficking cell wall components. J Integr Plant Biol.

[9] Clouse S (2011). Brassinosteroid signal transduction: from receptor kinase activation to transcriptional networks regulating plant development. Plant Cell.

[10] Rigó G, Ayaydin F, Tietz O, Zsigmond L, Kovács H, Páy A, Salchert K, Darula Z, Medzihradszky K, Szabados L, Palme K, Koncz C, Cséplo A (2013). Inactivation of plasma membrane-localized CDPK-RELATED KINASE5 decelerates PIN2 exocytosis and root gravitropic response in Arabidopsis. Plant Cell.

[11] Kleine-Vehn J, Friml J (2008). Polar targeting and endocytic recycling in auxin-dependent plant development. Annu Rev Cell Dev Biol.

[12] Wightman R, Turner S (2010). Trafficking of plant cellulose synthase complex. Plant Physiol.

[13] Symons G, Ross J, Jager C, Reid J (2008). Brassinosteroid transport. J Exp Bot.

[14] Cosgrove D (2005). Growth of the plant cell wall. Nat Rev Mol Cell Biol.

[15] Driouich A, Follet-Gueye M, Bernard S, Kousar S, Chevalier L, Vicré-Gibouin M, Lerouxel O (2012). Golgi-mediated synthesis and secretion of matrix polysaccharides of the primary cell wall of higher plants. Front Plant Sci.

[16] He B, Guo W (2009). The exocyst complex in polarized exocytosis. Curr Opin Cell Biol.

[17] Liu J, Guo W (2011). The exocyst complex in exocytosis and cell migration. Protoplasma.

[18] Heider M, Munson M (2012). Exorcising the exocyst complex. Traffic.

[19] Zárský V, Kulich I, Fendrych M, Pecenková T (2013). Exocyst complexes multiple functions in plant cells secretory pathways. Curr Opin Plant Biol.

[20] Zárský V, Cvrčková F, Potocký M, Hála M (2009). Exocytosis and cell polarity in plants – exocyst and recycling domains. New Phytol.

[21] Wu H, Rossi G, Brennwald P (2008). The ghost in the machine: small GTPases as spatial regulators of exocytosis. Trends in Cell Biol.

[22] Sivaram M, Saporita J, Furgason M, Boettcher A, Munson M (2005). Dimerization of the exocyst protein Sec6p and its interaction with the t-SNARE Sec9p. Biochemistry.

[23] Rivera-Molina F, Toomre D (2013). Live-cell imaging of exocyst links its spatiotemporal dynamics to various stages of vesicle fusion. J Cell Biol.

[24] Pečenková T, Hála M, Kulich I, Kocourková D, Drdová E, Fendrych M, Toupalová H, Žárský V (2011). The role for the exocyst complex subunits Exo70B2 and Exo70H1 in the plant-pathogen interaction. J Exp Bot.

[25] Cvrčková F, Grunt M, Bezvoda R, Hála H, Kulich I, Rawat S, Žárský V (2012). Evolution of the land plant exocyst complexes. Front Plant Sci.

[26] Zhang Y, Liu C, Emons A, Ketelaar T (2010). The Plant Exocyst. J Integr Plant Biol.

[27] Hála M, Cole R, Synek L, Drdová E, Pečenková T, Nordheim A, Lamkemeyer T, Madlung J, Hochholdinger F, Fowler J, Žárský V (2008). An exocyst complex functions in plant cell growth in arabidopsis and tobacco. Plant Cell.

[28] Cole R, Synek L, Žárský V, Fowler J (2005). SEC8, a subunit of the putative Arabidopsis exocyst complex, facilitates pollen germination and competitive pollen tube growth. Plant Physiol.

[29] Synek L, Schlager N, Eliáš M, Quentin M, Hauser M, Žárský V (2006). AtEXO70A1, a member of a family of putative exocyst subunits specifically expanded in land plants, is important for polar growth and plant development. Plant J.

[30] Fendrych M, Synek L, Pečenková T, Toupalová H, Cole R, Drdová E, Nebesářová J, Šedinová M, Hála M, Fowler J, Žárský V (2010). The Arabidopsis exocyst complex is involved in cytokinesis and cell plate maturation. Plant Cell.

[31] Li S, Chen C, Yu D, Ren S, Sun S, Liu L, Ketelaar T, Emons A, Liu C (2013). EXO70A1-mediated vesicle trafficking is critical for tracheary element development in Arabidopsis. Plant Cell.

[32] Wu J, Tan X, Wu C, Cao K, Li Y, Bao Y (2013). Regulation of cytokinesis by exocyst subunit SEC6 and KEULE in *Arabidopsis thaliana*. Mol Plant.

[33] Rybak K, Steiner A, Synek L, Klaeger S, Kulich I, Facher E, Wanner G, Kuster B, Zarsky V, Persson S, Assaad F (2014). Plant cytokinesis is orchestrated by the sequential action of the TRAPPII and Exocyst tethering complexes. Dev Cell.

[34] Fendrych M, Synek L, Pečenková T, Drdová E, Sekereš J, de Rycke R, Moritz K, Nowack M, Žárský V (2013). Visualization of the exocyst complex dynamics at the plasma membrane of *Arabidopsis thaliana*. Mole Biol Cell.

[35] Zhang Y, Immink R, Liu C, Emons A, Ketelaar T (2013). The Arabidopsis exocyst subunit SEC3A is essential for embryo development and accumulates in transient puncta at the plasma membrane. New Phytol.

[36] Drdová E, Synek L, Pečenková T, Hála M, Kulich I, Fowler J, Murphy A, Žárský V (2012). The exocyst complex contributes to PIN auxin efflux carrier recycling and polar auxin transport in Arabidopsis. Plant J.

[37] Lavy M, Bloch D, Hazak O, Gutman I, Poraty L, Sorek N, Sternberg H, Yalovsky S (2007). A novel ROP/RAC effector links cell polarity, root-meristem maintenance, and vesicle trafficking. Curr Biol.

[38] Hazak O, Bloch D, Poraty L, Sternberg H, Zhang J, Friml J, Yalovsky S (2010). A Rho scaffold integrates the secretory sytem with feedback mechanisms in regulation of auxin distribution. PLoS Biol.

[39] Van Damme D, Coutuer S, De Rycke R, Bouget F, Inzé D, Geelen D (2006). Somatic cytokinesis and pollen maturation in Arabidopsis depend on TPLATE, which has domains similar to coat proteins. Plant Cell.

[40] Moubayidin L, Perilli S, Ioio R, Di Mambro R, Costantino P, Sabatini S (2010). The rate of cell differentiation controls the Arabidopsis root meristem growth phase. Curr Biol.

[41] Verbelen J, De Cnodder T, Le J, Vissenberg K, Baluška F (2006). The root apex of Arabidopsis thaliana consists of four distinct zones of growth activities. Plant Signal Behav.

[42] Ding Z, Friml J (2010). Auxin regulates distal stem cell differentiation in Arabidopsis roots. Proc Natl Acad Sci U S A.

[43] Galinha C, Hofhuis H, Luijten M, Willemsen V, Blilou I, Heidstra R, Scheres B (2007). PLETHORA proteins as dose-dependent master regulators of Arabidopsis root development. Nature.

[44] Colón-Carmona A, You R, Haimovitch-Gal, Doerner P (1999). Spatio-temoral analysis of mitotic activity with a labile cyclin-GUS fusion protein. Plant J.

[45] Rahman A, Bannigan A, Sulaman W, Pechter P, Blancaflor E, Baskin T (2007). Auxin, actin, and growth of the Arabidopsis thaliana primary root. Plant J.

[46] Ivanov V, Dubrovsky J (1997). Estimation of the cell-cycle duration in the root apical meristem: a model of linkage between cell-cycle duration, rate of cell production, and rate of root growth. Int J Plant Sci.

[47] González-García M, Vilarrasa-Blasi J, Zhiponova M, Divol F, Mora-García S, Russinova E, Caño-Delgado A (2011). Brassinosteroids control meristem size by promoting cell cycle progression in *Arabidopsis* roots. Development.

[48] Hacham Y, Holland N, Butterfield C, Ubeda-Tomás S, Bennett M, Chory J, Savaldi-Goldstein S (2011). Brassinosteroid perception in the epidermis controls root meristem size. Development.

[49] Ritzenthaler C, Nebenführ A, Movafeghl A, Stussi-Garaud C, Behnia L, Pimpl P, Staehelin L, Robinson D (2002). Reevaluation of the effects of brefeldin A on plant cells using tobacco bright yellow 2 cells expressing Golgi-targeted green fluorescent protein and COPI antisera. Plant Cell.

[50] Robinson D, Langhans M, Saint-Jore-Dupas C, Hawes C (2008). BFA effects are tissue and not just plant specific. Trends Plant Sci.

[51] Driouich A, Zhang G, Staehelin L (1993). Effect of Brefeldin A on the structure of the Golgi apparatus and on the synthesis and secretion of proteins and polysaccharides in sycamore maple (Acer pseudoplatanus) suspension-cultured cells. Plant Physiol.

[52] Schindler T, Bergfeld R, Hohl M, Schopfer P (1994). Inhibition of Golgi-apparatus function by brefeldin A in maize coleoptiles and its consequences on auxin-mediated growth, cell-wall extensibility and secretion of cell-wall proteins. Planta.

[53] Lanubile R, Piro G, Dalessandro G (1997). Effect of Brefeldin A on the synthesis and transport of cell wall polysaccharides and proteins in pea root seedlings. J Exp Botany.

[54] Piro G, Montefussco A, Pacoda D, Dalessandro G (1999). Brefeldin A: a specific inhibitor of cell wall polysaccharide biosynthesis in oat coleoptiles segments. Plant Physiol Biochem.

[55] Baluška F, Hlavacka A, Šamaj J, Palme K, Robinson D, Matoh T, McCurdy D, Menzel D, Volkmann D (2002). F-actin dependent endocytosis of cell wall pectins in meristematic root cells. Insights from brefeldin A-induced compartments. Plant Physiol.

[56] Geldner N, Friml J, Stierhof Y, Jurgens G, Palme K (2001). Auxin transport inhibitors block PIN1 cycling and vesicle trafficking. Nature.

[57] Grebe M, Friml J, Swarup R, Ljung K, Sandberg G, Terlou M, Palme K, Bennett M, Scheres B (2002). Cell polarity signaling in Arabidopsis involves a BFA-sensitive auxin influx pathway. Current Biol.

[58] Takáč T, Pechan T, Richter H, Müller J, Eck C, Böhm N, Obert B, Ren H, Niehaus K, Šamaj J (2011). Proteomics on brefeldin a-treated Arabidopsis roots reveals profilin 2 as a new protein involved in the cross-talk between vesicular trafficking and the actin cytoskeleton. J Proteome Res.

[59] Feraru E, Feraru M, Asaoka R, Paciorek T, De Rycke R, Tanaka H, Nakano A, Friml J (2012). BEX5/RabA1b regulates trans-Golgi network-to-plasma membrane protein trafficking in Arabidopsis. Plant Cell.

[60] Blilou I, Xu J, Wildwater M, Willemsen V, Paponov I, Friml J, Heidstra R, Aida M, Plame K, Scheres B (2005). The PIN auxin efflux facilitator network controls growth and patterning in Arabidopsis roots. Nature.

[61] Saini S, Sharma I, Kaur N, Pati P (2013). Auxin: a master regulator in plant root development. Plant Cell Rep.

[62] Ioio R, Nakamura K, Moubayidin L, Perilli S, Taniguchi M, Morita M, Aoyama T, Costantino P, Sabatini S (2008). A genetic framework for the control of cell division and differentiation in the root meristem. Science.

[63] Chapman E, Estelle M (2009). Cytokinin and auxin intersection in root meristems. Genome Biol.

[64] Růžička K, Šimášková M, Duclercq J, Petrášek J, Zažímalová E, Simon S, Friml J, Van Montagu M, Benková E (2009). Cytokinin regulates root meristem activity via modulation of the polar auxin transport. Proc Natl Acad Sci U S A.

[65] Scacchi E, Salinas P, Gujas B, Santuari L, Krogan N, Ragni L, Berleth T, Hardtke C (2010). Spatio-temporal sequence of cross-regulatory events in root meristem growth. Proc Natl Acad Sci U S A.

[66] Moubayidin L, Mambro R, Sozzani R, Pacifici E, Salvi E, Terpstra I, Bao D, van Dijken A, Ioio R, Perilli S, Ljung K, Benfey P, Heidstra R, Costantino P, Sabatini S (2013). Spatial coordination between stem cell activity and cell differentiation in the root meristem. Dev Cell.

[67] Růžička K, Ljung K, Vanneste S, Podhorská R, Beeckman T, Friml J, Benková E (2007). Ethylene regulates root growth through effects on auxin biosynthesis and transport-dependent auxin distribution. Plant Cell.

[68] Swarup R, Perry P, Hagenbeek D, Van Der Straeten D, Beemster G, Sandberg G, Bhalerao R, Ljung K, Bennet M (2007). Ethylene upregulates auxin biosynthesis in Arabidopsis seedlings to enhance inhibition of root cell elongation. Plant Cell.

[69] Strader L, Chen G, Bartel B (2010). Ethylene directs auxin to control root cell expansion. Plant J.

[70] Lewis D, Negi S, Sukumar P, Muday G (2011). Ethylene inhibits lateral root development, increases IAA transport and expression of PIN3 and PIN7 auxin efflux carriers. Development.

[71] Stepanova A, Yun J, Likhacheva A, Alonso J (2007). Multilevel interactions between ethylene and auxin in Arabidopsis roots. Plant Cell.

[72] Jaillais Y, Fobis-Loisy I, Miège C, Rollin C, Gaude T (2006). AtSNX1 defines an endosome for auxin-carrier trafficking in Arabidopsis. Nature.

[73] Kleine-Vehn J, Dhonukshe P, Swarup R, Bennett M, Friml J (2006). Subcellular trafficking of the Arabidopsis auxin influx carrier uses a novel pathway distinct from PIN1. Plant Cell.

[74] Robert S, Narasimha Chary S, Drakakaki G, Li S, Yang Z, Raikhel N, Hicks G (2008). Endosidin1 defines a compartment involved in endocytosis of the brassinosteroid receptor BRI1 and the auxin transporters PIN2 and AUX1. Proc Natl Acad Sci U S A.

[75] Qi X, Kaeda M, Chen J, Geitmann A, Zheng H (2011). A specific role for Arabidopsis TRAPPII in post-Golgi trafficking that is crucial for cytokinesis and cell polarity. Plant J.

[76] Langowski L, Růžička K, Naramoto S, Kleine-Vehn J, Friml J (2010). Trafficking to the outer polar domain defines the root-soil interface. Curr Biol.

[77] Sassi M, Lu Y, Zhang Y, Wang J, Dhonukshe P, Blilou I, Dai M, Li J, Ximing G, Jaillais Y, Yu X, Traas J, Ruberti I, Wang H, Scheres B, Vernoux T, Xu J (2012). COP1 mediates the coordination of root and shoot growth by light through modulation of PIN1- and PIN2-dependent auxin transport in Arabidopsis. Development.

[78] Wan Y, Jasik J, Wang L, Hao H, Volkmann D, Menzel D, Mancuso S, Baluška F, Lin J (2012). The signal transducer NPH3 integrates the Phototropin1 photosensor with PIN2-based polar auxin transport in Arabidopsis root phototropism. Plant Cell.

[79] Stahl Y, Wink R, Ingram G, Simon R (2009). A signaling module controlling the stem cell niche in Arabidopsis root meristems. Curr Biol.

[80] Wang Z, Bai M, Oh E, Zhu J (2012). Brassinosteroid signaling network and regulation of photomorphogenesis. Annu Rev Genet.

[81] Fridman Y, Savaldi-Goldstein S (2013). Brassinosteroids in growth control: how, when, and where. Plant Sci.

[82] Tang B, Kim T, Oses-Prieto J, Sun Y, Deng Z, Zhu S, Wang R, Burlingame A, Wang Z (2008). BSKs mediate signal transduction from the receptor kinase BRI1 in Arabidopsis. Science.

[83] Mani R, St Onge R, Harman J, Glaever G, Roth F (2008). Defining genetic interaction. Proc Natl Acad Sci U S A.

[84] Phillips P (2008). Epistasis – the essential role of gene interactions in the structure and evolution of genetic systems. Nat Rev Genet.

[85] Buschmann H, Lloyd C (2008). Arabidopsis mutants and the network of microtubule-associated functions. Mol Plant.

[86] Wang Z, Nakano T, Gendron J, He J, Chen M, Vafaeados D, Yang Y, Fujioka S, Yoshida S, Asami T, Chory J (2002). Nuclear-localized BZR1 mediates brassinosteroid-induced growth and feedback suppression of brassinosteroid biosynthesis. Dev Cell.

[87] Tanaka K, Asami T, Yoshida S, Nakamura Y, Matsuo T, Okamoto S (2005). Brassinosteroid homeostasis in Arabidopsis is ensured by feedback expressions of multiple genes involved in its metabolism. Plant Physiol.

[88] Scacchi E, Osmont K, Beauchat J, Salinas P, Navarrete-Gómez M, Trigueros M, Ferrándiz C, Hardtke C (2009). Dynamic, auxin-responsive plasma membrane-to-nucleus movement of Arabidopsis BRX. Development.

[89] Santuari L, Scacchi E, Rodriguez-Villalon A, Salinas P, Dohmann E, Grunoud G, Vemoux T, Smith R, Hardtke C (2011). Positional information by differential endocytosis splits auxin response to drive Arabidopsis root meristem growth. Curr Biol.

[90] Mouchel C, Osmont K, Hardtke C (2006). BRX mediates feedback between brassinosteroid levels and auxin signaling in root growth. Nature.

[91] Mouchel C, Briggs G, Hardtke C (2004). Natural genetic variation in Arabidopsis identifies BREVIS RADIX, a novel regulator of cell proliferation and elongation in the root. Genes Dev.

[92] Gujas B, Alonso-Blanco C, Hardtke C (2012). Natural Arabidopsis brx loss-of-function alleles confer root adaptation to acidic soil. Curr Biol.

[93] Kubesˇ M, Yang H, Richter G, Cheng Y, Młodzińska E, Wang X, Blakeslee J, Carraro N, Petrášek J, Zažímalová E, Hoyerová K, Peer W, Murphy A (2011). The Arabidopsis concentration-dependent influx/efflux transporter ABCB4 regulates cellular auxin levels in the root epidermis. Plant J.

[94] Lin R, Wang H (2005). Two homologous ATP-binding cassette transporter proteins, AtMDR1 and AtPGP1, regulate Arabidopsis photomorphogenesis and root development by mediating polar auxin transport. Plant Physiol.

[95] Lewis D, Miller N, Splitt B, Wu G, Spalding E (2007). Separating the roles of acropetal and basipetal auxin transport on gravitropism with mutations in two Arabidopsis multidrug resistance-like ABC transporter genes. Plant Cell.

[96] Terasaka K, Blakeslee J, Titapiwatanakun B, Peer W, Bandyopadhyay A, Makam S, Lee O, Richards E, Murphy A, Sato F, Yazaki K (2005). PGP4, an ATP binding cassette P-glycoprotein, catalyzes auxin transport in Arabidopsis thaliana roots. Plant Cell.

[97] Wu G, Lewis D, Spalding E (2007). Mutations in Arbidopsis multidrug resistance-like ABC transporters separate the roles of acropetal and basipetal auxin transport in lateral root development. Plant Cell.

[98] Zažímalová E, Murphy A, Yang H, Hoyerová K, Hošek P (2010). Auxin transporters – why so many?. Cold Spring Harb Perspect Biology.

[99] Sauer M, Robert S, Klein-Vehn J (2013). Auxin: simply complicated. J Exp Bot.

[100] Caesar K, Elgass K, Chen Z, Huppenberger P, Witthoft J, Schleifenbaum F, Blatt M, Oecking C, Harter K (2011). A fast brassinolide-regulated response pathway in the plasma membrane of Arabidopsis thaliana. Plant J.

[101] Zhao Y, Qi Z, Berkowitz G (2013). Teaching an old hormone new tricks: cytosolic Ca^2+^ elevation involvement in plant brassinosteroid signal transduction cascades. Plant Physiol.

[102] Zhang X, Wang P, Akanksha G, Zhang J, Brennwald P, TerBush D, Guo W (2005). Lethal giant larvae proteins interact with the exocyst complex and are involved in polarized exocytosis. J Cell Biol.

[103] Tsukagoshi H, Busch W, Benfey P (2010). Transcriptional regulation of ROS controls transition from proliferation to differentiation in the root. Cell.

[104] Liu Y, Lai N, Gao K, Chen F, Yuan L, Mi G (2013). Ammonium inhibits primary root growth by reducing the length of meristem and elongation zone and decreasing elemental expansion rate in the root apex in Arabidopsis thaliana. PLoS One.

[105] Markakis M, Cnodder T, Lewandowski M, Simon D, Boron A, Balcerowicz D, Doubbo T, Taconnat L, Renou J, Hofte H, Verbelen J, Vissenberg K (2012). Identification of genes involved in the ACC-mediated control of root cell elongation in Arabidopsis. BMC Plant Biol.

[106] Niu Y, Chai R, Jin G, Wang H, Tang C, Zhang Y (2013). Responses of root architecture development to low phosphorus availability: a review. Ann Bot.

[107] Baskin T (2013). Patterns of root growth acclimation: constant processes, changing boundaries. WIRE Dev Biol.

[108] Harberd N, Belfield E, Yasumura Y (2009). The angiosperm gibberelling-GID1-DELLA growth regulatory mechanism: how an “inhibitor of an inhibitor” enables flexible response to fluctuating environments. Plant Cell.

[109] Vanneste S, Friml J (2009). Auxin: a trigger for change in plant development. Cell.

[110] Suzuki N, Koussevitzky S, Mittler R, Miller G (2012). ROS and redox signaling in the response of plants to abiotic stress. Plant Cell Environ.

[111] Baxter A, Mittler R, Suzuki N (2014). ROS as key players in plant stress signaling. J Exp Botany.

[112] Robert S, Bichet A, Grandjean O, Kierzkowski D, Satiat-Jeunemaitre B, Pelletier S, Hauser M, Hofte H, Vernhettes S (2005). An Arabidopsis endo1,4-β-gluconase involved in cellulose synthesis undergoes regulated intracellular cycling. Plant Cell.

[113] Vissenberg K, Oyama M, Osato Y, Yokoyama R, Verbelen J, Nishitani K (2005). Differential expression of AtXTH17, AtXTH18, AtXTH19, and AtXTH20, genes in Arabidopsis roots. Physiological roles in specification in cell wall construction. Plant Cell Physiol.

[114] Osato Y, Yokoyama R, Nishitani K (2006). A principal role for AtXTH18 in Arabidopsis thaliana root growth: a functional analysis using RNAi plants. J Plant Res.

[115] Passardi F, Tognolli M, De Meyer M, Penel C, Dunand C (2006). Two cell wall associated peroxidases from Arabidopsis influence root elongation. Planta.

[116] Guo W, Zhao J, Li X, Qin L, Yan X, Liao H (2011). A soybean b-expansin gene GmEXPB2 intrinsically involved in root system architecture responses to abiotic stresses. Plant J.

[117] Zhang J, Xu L, Wu Y, Chen X, Liu Y, Zhu S, Ding W, Wu P, Yi K (2012). OsGLU3, a putative membrane-bound endo-1,4-beta-glucanase, is required for root cell elongation and division in rice (Oryza sativa L.). Mol Plant.

[118] Ubeda-Tomás S, Beemster G, Bennett M (2012). Hormonal regulation of root growth: integrating local activities into global behavior. Trends Plant Sci.

[119] Alonso JM, Stepanova AN, Leisse TJ, Kim CJ, Chen H, Shinn P, Stevenson DK, Zimmerman J, Barajas P, Cheuk R, Gadrinab C, Heller C, Jeske A, Koesema E, Meyers CC, Parker H, Prednis L, Ansari Y, Choy N, Deen H, Geralt M, Hazari N, Hom E, Karnes M, Mulholland C, Ndubaku R, Schmidt I, Guzman P, Aguilar-Henonin L, Schmid M (2003). Genome-wide insertional mutagenesis of Arabidopsis thaliana. Science.

[120] Rosso MG, Li Y, Strizhov N, Reiss B, Dekker K, Weisshaar B (2003). An Arabidopsis thaliana T-DNA mutagenized population (GABI-Kat) for flanking sequence tag-based reverse genetics. Plant Mol Biol.

[121] Malamy J, Benfey P (1997). Organization and cell differentiation in lateral roots of Arabidopsis thaliana. Development.

[122] Ivanchenko M, Coffeen W, Lomax T, Dubrovsky J (2006). Mutations in the Digeotropica (Dgt) gene uncouple patterned cell division during lateral root initiation form proliferative cell division in the pericycle. Plant J.

[123] Silk W, Lord E, Eckard K (1989). Growth patterns inferred from anatomical records: empirical tests using longisections of roots in Zea mays L. Plant Physiol.

[124] Rymen B, Coppens F, Dhondt S, Fiorani F, Beemster G, Hennic L, Kohler C (2010). Kinematic Analysis of Cell Division and Expansion. Plant Developmental Biology, Methods in Molecular Biology.

[125] Band L, Ubeda-Tomás S, Dyson R, Middleton A, Hodgman T, Owen M, Jensen O, Bennett M, King J (2012). Growth-induced hormone dilution can explain the dynamics of plant root cell elongation. Proc Natl Acad Sci U S A.

[126] Ron M, Dorrity M, de Lucas M, Toal T, Hernandez R, Little S, Maloof J, Kliebensten D, Brady S (2013). Identification of novel loci regulating interspecific variation in root morphology and cellular development in Tomato. Plant Physiol.

[127] Czechowski T, Stitt M, Altmann T, Udvardi M, Scheible W (2005). Genome-wide identification and testing of superior reference genes for transcript normalization in Arabidopsis. Plant Physiol.

[128] Expósito-Rodríguez M, Borges A, Borges-Pérez A, Pérez J (2008). Selection of internal control genes for quantitative real-time RT-PCR studies during tomato development process. BMC Plant Biol.

[129] Remans T, Smeets K, Opdenakker K, Mathijsen D, Vangronsveld J, Cuypers A (2008). Normalization of real-time RT-PCR gene expression measurements in Arabidopsis thaliana exposed to increased metal concentrations. Planta.

[130] Rieu I, Eriksson S, Powers S, Gong F, Griffiths J, Woolley L, Benlloch R, Nilsson O, Thomas S, Hedden P, Phillips A (2008). Genetic analysis reveals that C19-GA 2-oxidation is a major gibberllin inactivation pathway in Arabidopsis. Plant Cell.

[131] Tromas A, Braun N, Muller P, Khodus T, Paponov I, Palme K, Ljung K, Lee J, Benfey P, Murray J, Scheres B, Perrot-Rechenmann C (2009). The AUXIN BINDING PROTEIN 1 is required for differential auxin responses mediating root growth. PLoS One.

[132] Hellemans J, Mortier G, De Paepe A, Speleman F, Vandesompele J (2007). qBase relative quantification framework and software for management and automated analysis of real-time quantitative PCR data. Genome Biol.

