# Supplementary material for: Developmentally distinct activities of the exocyst enable rapid cell elongation and determine meristem size during primary root growth in Arabidopsis
Source: BMC Plant Biol. 2014 Dec 31;14:386. doi: 10.1186/s12870-014-0386-0 (PMC4302519; doi:10.1186/s12870-014-0386-0)
Supplement: Additional file 1: — Supplemental Figures. Figure S1 - Exocyst mutant seedling images; Figure S2 - Plethora-YFP in mutant root tips; Figure S3 - T-test comparisons of root growth characteristics; Figure S4 - Phytohormone dose-response curves of exocyst mutants; Figure S5 - Fluorescently labeled auxin transporters in sec8-4 exo70A1 root tips; Figure S6 - Fluorescently labeled auxin transporters in sec8-3 root tips; Figure S7 - pDR5-GUS in dark and light grown seedling root tips; Figure S8 - Lugol-stained starch in root tips of exocyst mutants; Figure S9 - BRI1-GFP in mutant root tips and epibrassinolide dose-response; Figure S10 - Effect of BSK3 overexpression on root growth in exocyst mutants; Figure S11 - Effect of BFA on root growth in exocyst mutants. [file 12870_2014_386_MOESM1_ESM.pdf]

Additional File 1

Supplemental Figures:

Figure S1 Exocyst mutant seedling images

Figure S2 Plethora-YFP in mutant root tips;

Figure S3 t-Test comparisons of root growth characteristics;

Figure S4 Dose-response curves of exocyst mutants to phytohormones;

Figure S5 Fluorescently labeled auxin transporters in *sec8-4 exo70A1* root tips;

Figure S6 Fluorescently labeled auxin transporters in *sec8-3* root tips;

Figure S7 pDR5-GUS in dark and light grown seedling root tips

Figure S8 Lugol-stained starch in root tips of exocyst mutants

Figure S9 BRI1-GFP in mutant root tips and epibrassinolide dose-response;

Figure S10 Effect of BSK3 overexpression on root growth in exocyst mutants;

Figure S11 Root growth sensitivity of exocyst mutants to manipulations that alter BRX mediated signaling

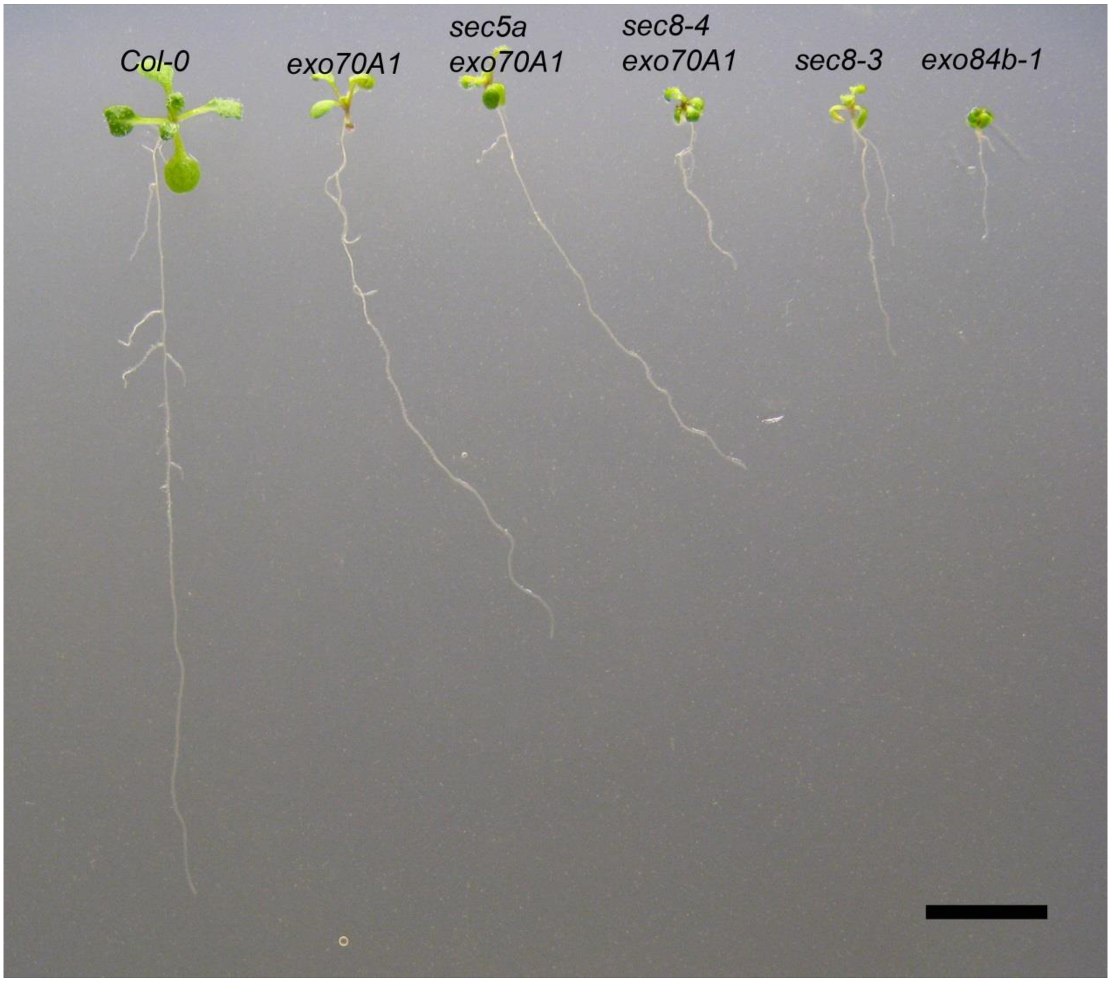

Figure S1. Exocyst mutants are dwarf and have shorter roots.
Shown are 8-day-old seedlings grown on a vertically oriented plate. From left to right are *Col0, exo70A1, sec5a exo70A1, sec8-4 exo70A1, sec8-3*, and *exo84b-1* seedlings. Plants with *exo70A1* show skewing to the right as previously reported. Bar = 10 mm.

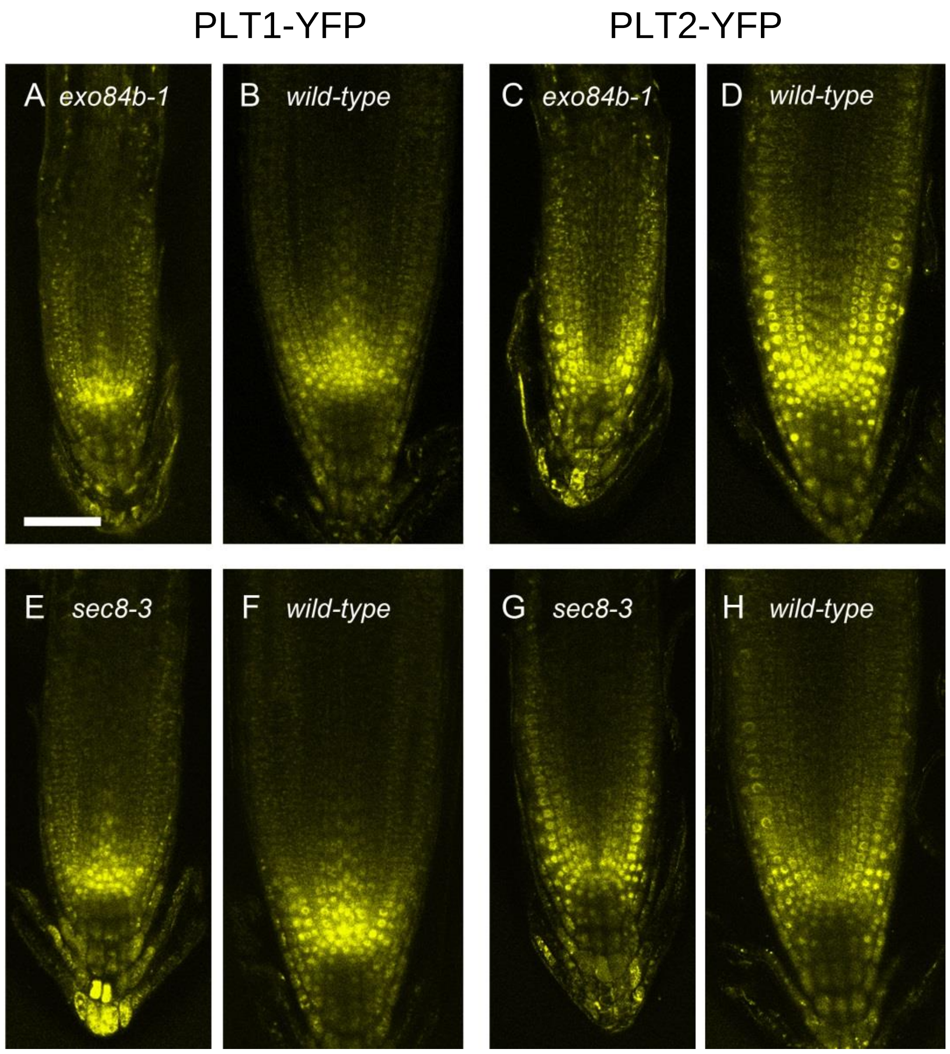

Figure S2. Expression patterns of *PLT1-YFP* and *PLT2-YFP* in the root tips of seedlings homozygous for *exo84b-1* or *sec8-3* are similar to the patterns observed in their wild-type siblings. (size bar = 50 µm).

Exocyst mutants	*exo70A1*	*sec5a exo70A1*	*sec8-4 exo70A1*	*sec8-3*	*exo84b*
Root Growth Rate	<0.001	<0.001	<0.001	<0.001	<0.001
Cell Production Rate	0.018	<0.001	<0.001	<0.001	<0.001
Meristem Length (# cells)	<0.001	<0.001	<0.001	<0.001	<0.001
Length of Cell Cycle	0.050	0.476	0.062	0.169	0.001
Mature Cortical Cell Length	0.044	0.065	<0.001	<0.001	<0.001
Elongation Zone Length (# cells)	0.042	0.003	<0.001	<0.001	<0.001
Time in Elongation Zone	0.065	<0.001	0.004	<0.001	<0.001
Elongation Rate Constant	<0.001	<0.001	<0.001	<0.001	<0.001
Mature Cortical Cell Width	0.014	<0.001	<0.001	<0.001	<0.001
Root Diameter (elongation zone)	<0.001	<0.001	<0.001	<0.001	<0.001
Mature Cortical Cell Volume	<0.001	<0.001	<0.001	<0.001	<0.001
Mature Cell Length/Width	<0.001	<0.001	<0.001	<0.001	<0.001

Comparitors	*bri1*	*det2-1*	*pin2-1*	*aux1-7*	*BFA tx 10mM*
Root Growth Rate	<0.001	<0.001	<0.001	<0.001	<0.001
Cell Production Rate	<0.001	<0.001	<0.001	<0.001	<0.001
Meristem Length (# cells)	0.250	0.015	<0.001	<0.001	<0.001
Length of Cell Cycle	<0.001	<0.001	0.483	0.449	0.100
Mature Cortical Cell Length	<0.001	<0.001	0.366	0.131	<0.001
Elongation Zone Length (# cells)	<0.001	<0.001	<0.001	<0.001	<0.001
Time in Elongation Zone	0.094	0.057	0.017	<0.001	0.456
Elongation Rate Constant	<0.001	<0.001	<0.001	<0.001	<0.001
Mature Cortical Cell Width	<0.001	<0.001	<0.001	<0.001	0.354
Root Diameter (elongation zone)	<0.001	<0.001	<0.001	<0.001	<0.001
Mature Cortical Cell Volume	<0.001	<0.001	<0.001	<0.001	<0.001
Mature Cell Length/Width	<0.001	<0.001	<0.001	<0.001	<0.001

Figure S3. Table of *p* values for one-sided t-Test comparison of growth parameters in mutant lines compared to Col-0, for data in Figure 2 of main text. Most comparisons are between seven roots of each genotype, but mature cortical cell widths, volumes, and length/width ratios compare a large sample of individual cells from each genotype (measured from each of seven roots), so that n=71-152. Highlighted in grey are values indicating an insignificant difference from Col-0 (p>0.05). The p values shown for the elongation rate constant were obtained from a multiple regression analysis, rather than a t-Test.

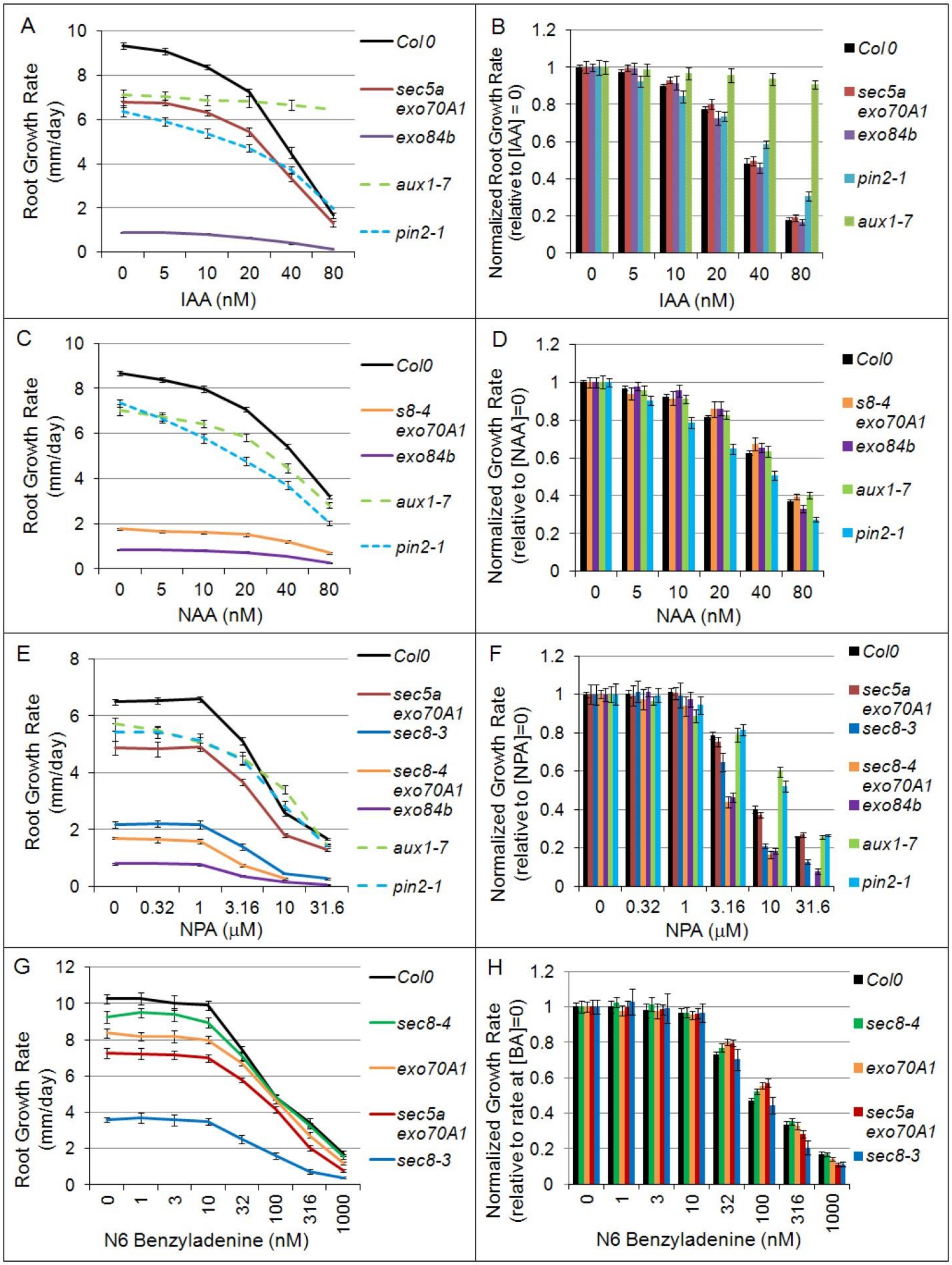
A
B
C
D
E
F
G
H
Root Growth Rate (mm/day)
Normalized Root Growth Rate (relative to [IAA] = 0)
Normalized Growth Rate (relative to [NAA]=0)
Normalized Growth Rate (relative to [NPA]=0)
Normalized Growth Rate (relative to rate at [BA]=0)
IAA (nM)
NAA (nM)
NPA (μM)
N6 Benzyladenine (nM)
Col0
sec5a exo70A1
exo84b
aux1-7
pin2-1
s8-4 exo70A1
sec8-3
sec8-4 exo70A1
sec8-4
exo70A1

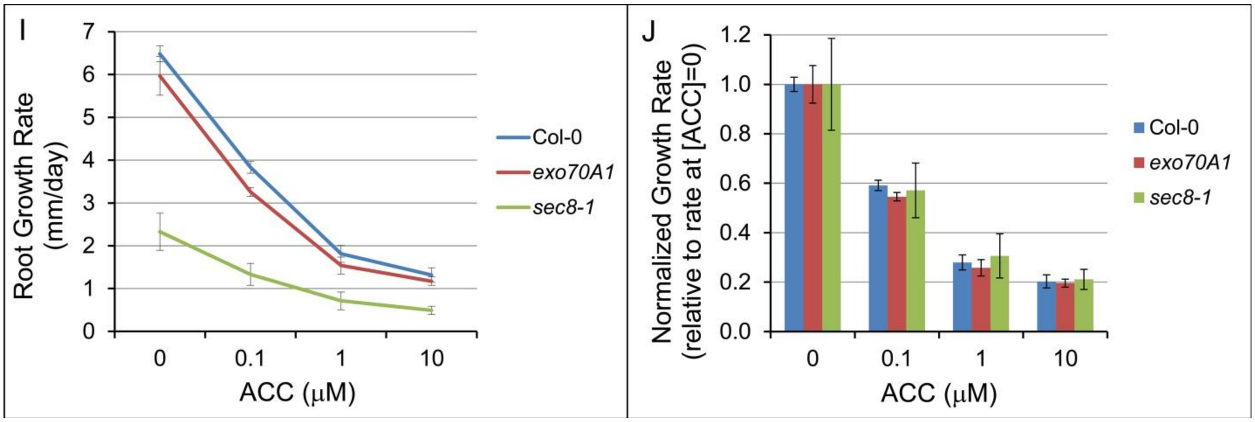

Figure S4. Primary root growth response of exocyst mutants to auxin, cytokinin, and ACC treatments is similar to wild-type. Second column graphs (B, D, F, H and J) present data normalized to the root growth rate when no hormone has been added (error bars = standard error).

IAA (A and B): The normalized dose-responses of exocyst mutants *sec5a exo70* and *exo84b-1* are not significantly different from Columbia 0 ($p>0.05$, t-test, n=28-30 for each genotype at each concentration). Similar results were obtained in a separate experiment for an *exo70A1-2* single mutant (data not shown). In contrast, the *aux1-7* mutant demonstrates insensitivity to IAA with significantly higher normalized growth rates compared to Col-0 at IAA concentrations greater than 5 nM ($p<0.05$, t-test, n=28-30). The *pin2-1* mutant also demonstrates an altered dose response to IAA compared with Col-0. The normalized growth rate for *pin2-1* was slightly lower than Col-0 when treated with 10 nM IAA ($p<0.05$, t-test, n=30) and slightly greater than Col-0 when treated with 40 or 80 nM IAA ($p<0.05$, t-test, n=29-30).

NAA (C and D): The normalized growth rates for *sec8-4 exo70*, *exo84b-1*, and *aux1-7* were not significantly different from wild-type Col-0 at all NAA concentrations tested ($p>0.05$, t-test, n=27-30). In contrast, the normalized growth rates for *pin2-1* seedlings were significantly different at all concentrations of NAA evaluated ($p<0.015$, t-test, n=29-30). Because NAA enters cells without the aid of a transporter, the import transport mutant, *aux1-7*, was expected to have a normalized dose response curve that was similar to Col-0, and this was observed ($p>0.05$, t-test, n=28-30).

NPA (E and F): The shape of the root growth rate response curves following treatment with the auxin transport inhibitor, NPA, is similar for both exocyst mutants and Col-0 (E). These curves demonstrate a precipitous fall in growth rate at NPA concentrations above 1 micromolar. When normalized, the dose response of the *sec5a exo70* mutant does not differ significantly from Col-0 ($p>0.05$, t-test, n=23-27 at each concentration). The normalized data for exocyst mutants with more severe root growth defects, *sec8-3, sec8-4 exo70A1-2,* and *exo84b-1*, indicate increased sensitivity to NPA concentrations above 1 □M compared to Col-0 ($p<0.01$, t-test, n=20-28). In contrast, the auxin transport mutants *aux1-7* and *pin2-1* have NPA growth response curves that are different in shape than Col-0. This difference is accentuated in the normalized data, which show the auxin transport mutants to have significantly decreased sensitivity to NPA at 10 □M ($p<0.01$, t-test, n=23-24).

N6-Benzyladenine (G and H): Both exocyst mutants and Col-0 show a similar root growth response to treatment with this cytokinin, with decreased growth rates observed at concentrations greater than 10 nM. The normalized growth rates at 31.6 and 100 nM for both *exo70A1* and *sec5a exo70A1* were slightly, but statistically higher than Col-0 ($p<0.01$, t-test, n=24), whereas at 316 and 1000 nM the normalized growth rates were slightly lower than Col-0 for *sec5a exo70A1* and *sec8-3* ($p<0.01$, t-test, n=13-24). These small differences do not correlate with the degree of growth defect observed in the various exocyst mutants, suggesting that they are not directly linked to exocyst function.

ACC (I and J). The normalized growth rate response to the ethylene precursor ACC was similar in *sec8-1* and *exo70A1-2*, compared to Col-0, at all concentrations evaluated ($p>0.05$, t-test, n=10-12).

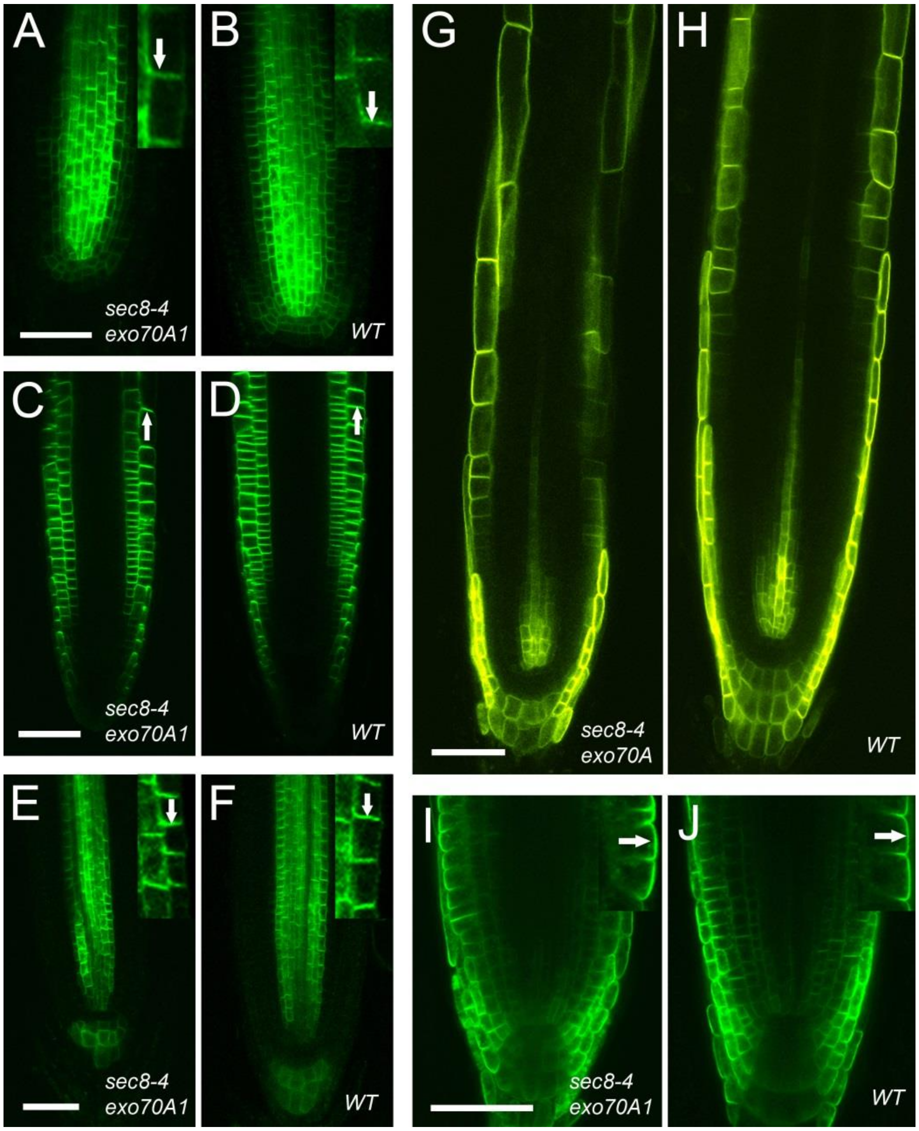

Figure S5. Auxin transport proteins in the root tips of *sec8-4 exo70A1* (A, C, E, G, and I) are expressed and localized within root tip cells in patterns similar to that of their wild-type siblings (B, D, F, H, and J). A and B: PIN1-GFP. C and D: PIN2-YFP. E and F: PIN7-GFP. G and H: AUX1-YFP. I and J: ABCG36-GFP. Insets are higher magnifications of cells in the larger image; arrows point to examples of polarized localization. Bars= 50 microns.

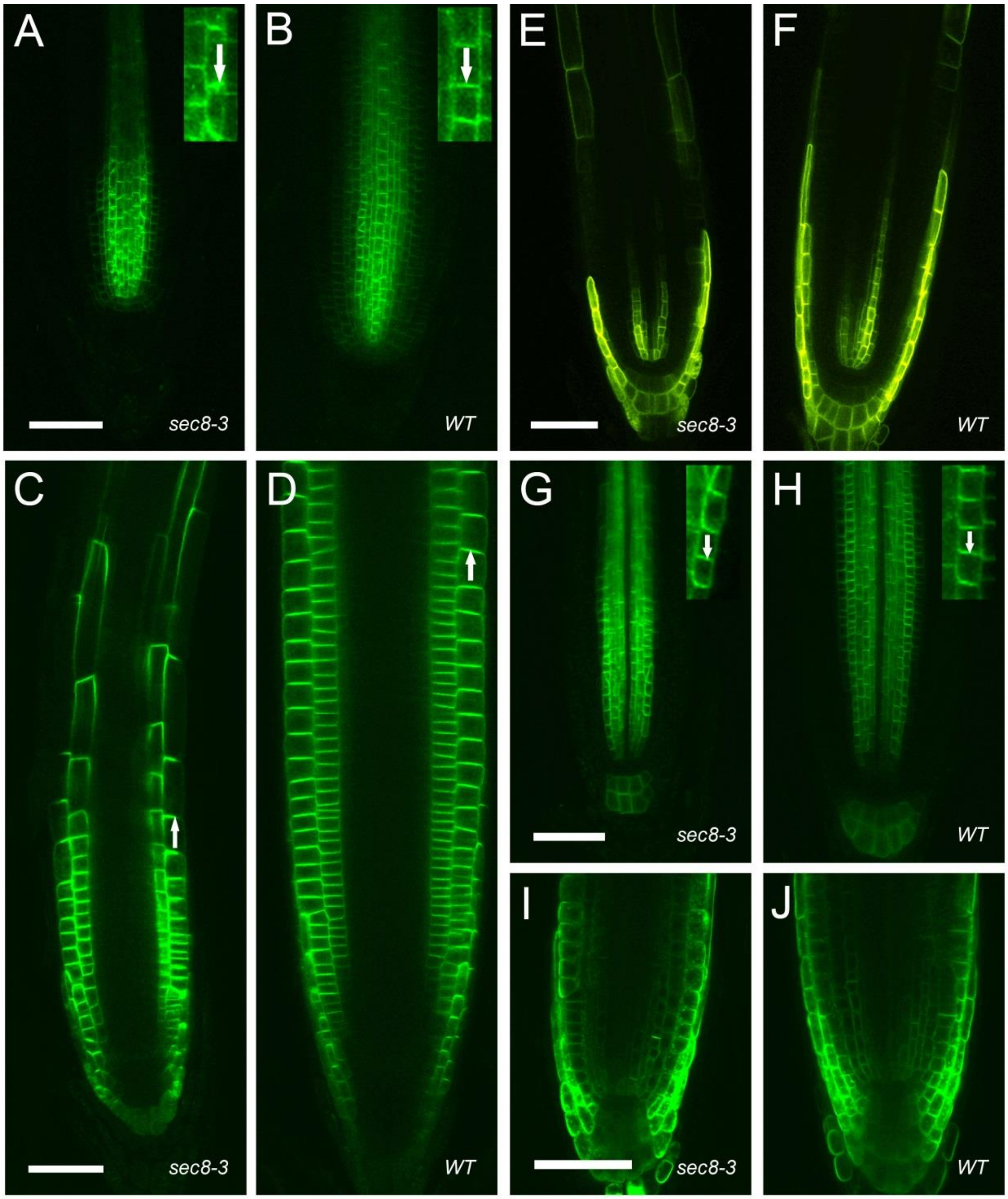

Figure S6. Auxin transport proteins in the root tips of *sec8-3* mutants (A, C, E, G, and I) are expressed and localized in a pattern similar to that of their wild-type siblings (B, D, F, H, and J). A and B: PIN1-GFP. C and D: PIN2-GFP. E and F: AUX1-YFP. G and H: PIN7-GFP. I and J: ABCG36-GFP. Insets are higher magnifications of cells in larger image; arrows point to examples of polarized localization. Bars= 50 microns.

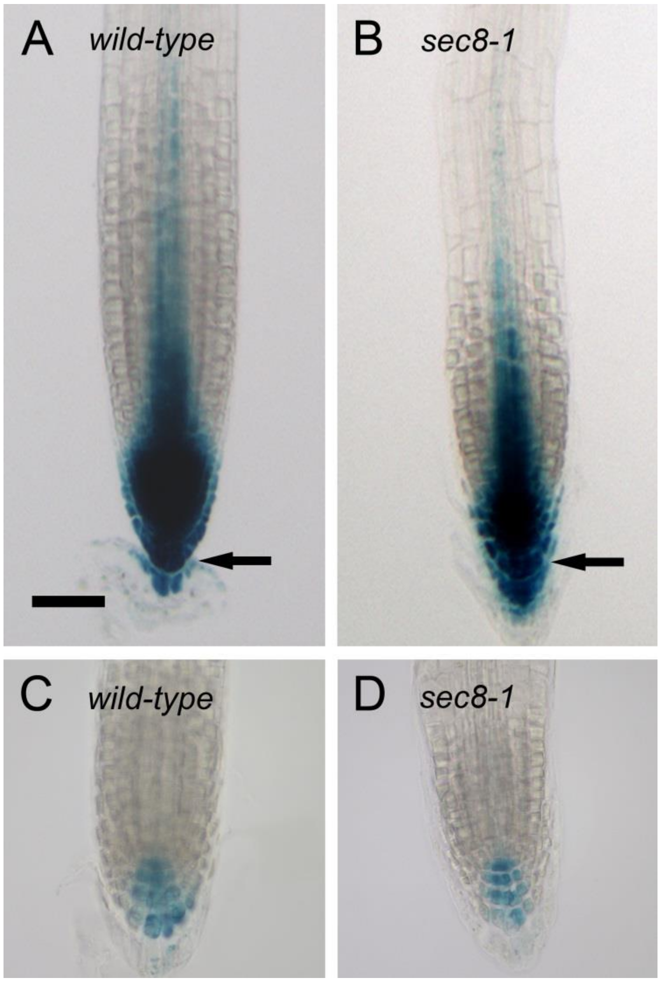

Figure S7. DR5-auxin response marker in root tips of light and dark grown seedlings. Seedlings containing a *pDR5:GUS* marker for auxin response were grown for 5 days in the light (A,B) or dark (C,D). In light-grown seedlings, staining for the GUS marker showed high expression focused at the quiescent center and columella cells in both *sec8-1* mutants (B) and their wild-type siblings (A). Arrows point to comparable layers of columella cells, with the outer layers of the root cap showing a reduced tendency to slough off in the mutant. In dark grown seedlings (C and D), auxin response was significantly reduced, consistent with previous work demonstrating reduced auxin production and reduced acropetal auxin transport in dark grown seedlings (Sassi, et al., 2012; Wan, et al., 2012). In dark grown seedlings the pattern of expression, located predominantly in the columella, was similar in both *sec8-1* mutants (D) and their wild-type siblings (C). Bar = 50 µm.

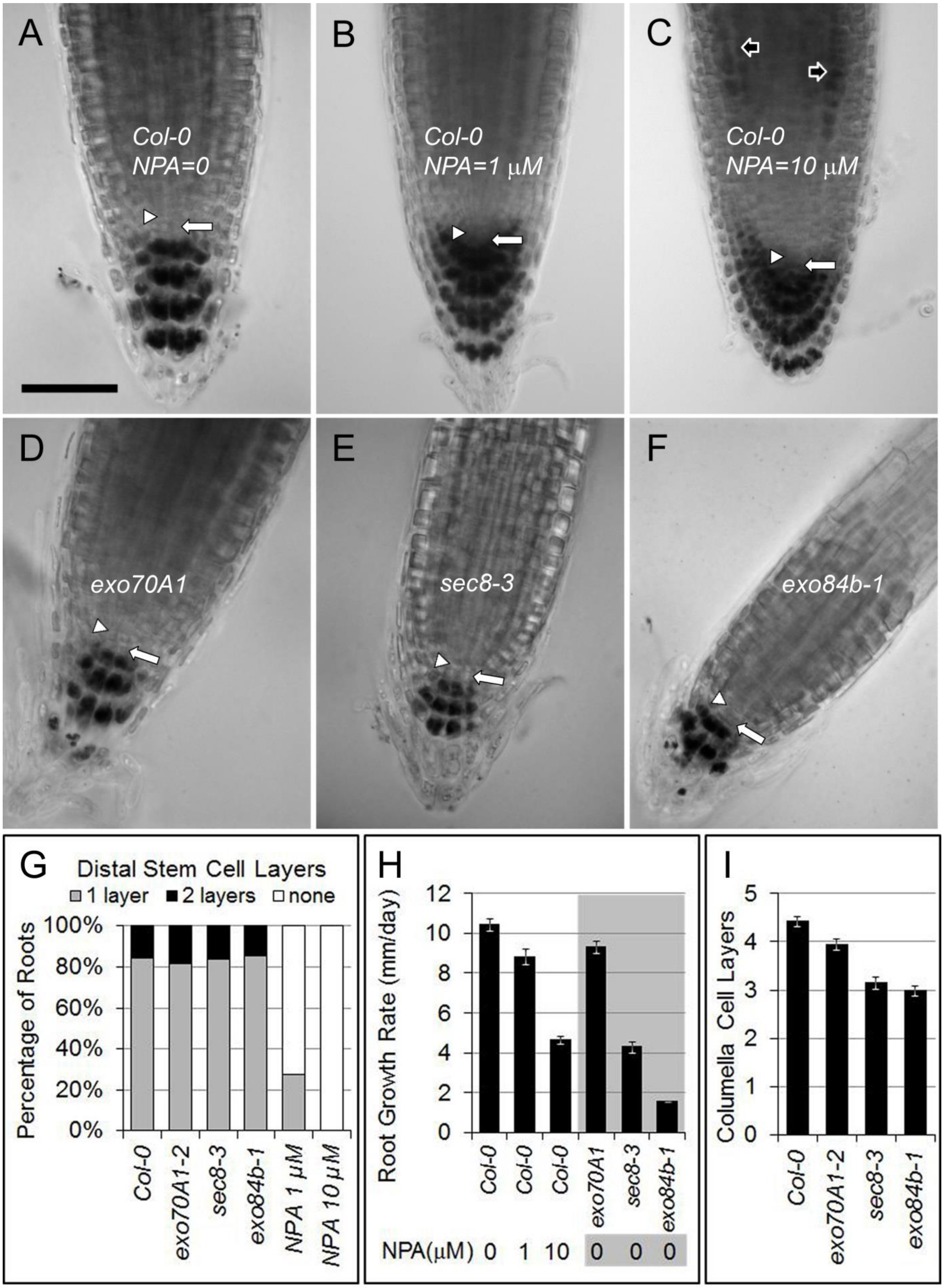

A
Col-0
NPA=0
B
Col-0
NPA=1 μM
C
Col-0
NPA=10 μM
D
exo70A1
E
sec8-3
F
exo84b-1
G
Distal Stem Cell Layers
1 layer
2 layers
none
Percentage of Roots
100%
80%
60%
40%
20%
0%
Col-0
exo70A1-2
sec8-3
exo84b-1
NPA 1 μM
NPA 10 μM
H
Root Growth Rate (mm/day)
12
10
8
6
4
2
0
Col-0
Col-0
Col-0
exo70A1
sec8-3
exo84b-1
NPA(μM) 0 1 10 0 0 0
I
Columella Cell Layers
5
4
3
2
1
0
Col-0
exo70A1-2
sec8-3
exo84b-1

Figure S8. Lugol-stained starch in root tips of exocyst mutants (previous page)
Starch-containing amyloplasts appear darkly stained in the differentiated columella cells of seven day old seedlings after Lugol staining (n>20 roots of each genotype or treatment) (A-F). Starch is not detected in the quiescent center (white triangles) or undifferentiated distal stem cells (DSC) (white arrows) of *exo70A1* (D), *sec8-3* (E) and *exo84b-1* (F) mutant root tips, a pattern similar to that in Col-0 (A). This contrasts with the staining pattern in positive controls, where auxin transport is inhibited in Col-0 roots by growth on media containing NPA (B, C). In B (NPA=1 μM), starch appears more broadly distributed in the root tip, such that undifferentiated DSC cells are not identified. In C (NPA=10 μM), the expanded region of starch includes cells in the elongation zone (black arrows). Bar (A-F) = 50 μm. When the number of undifferentiated (i.e. starchless) DSC cell layers are quantified for each root (G), approximately 80% of the exocyst mutant root tips have 1 layer of DSCs between the quiescent center and Lugol-stained columella, with the remaining 20% having 2 such layers, similar to Col-0, but contrasting with the NPA-grown controls (G). The defect in root growth rates observed in two exocyst mutants, *sec8-3* and *exo84b-1,* is at least as severe as that observed following treatment with 10μM NPA (H). Although the DSC layer pattern is similar to wild-type, the exocyst mutants demonstrate significantly fewer layers of starch-stained columella cells (p<0.0, t-test, n>20 for each genotype) (I), consistent with the overall trend observed for shorter developmental zones in the mutants.

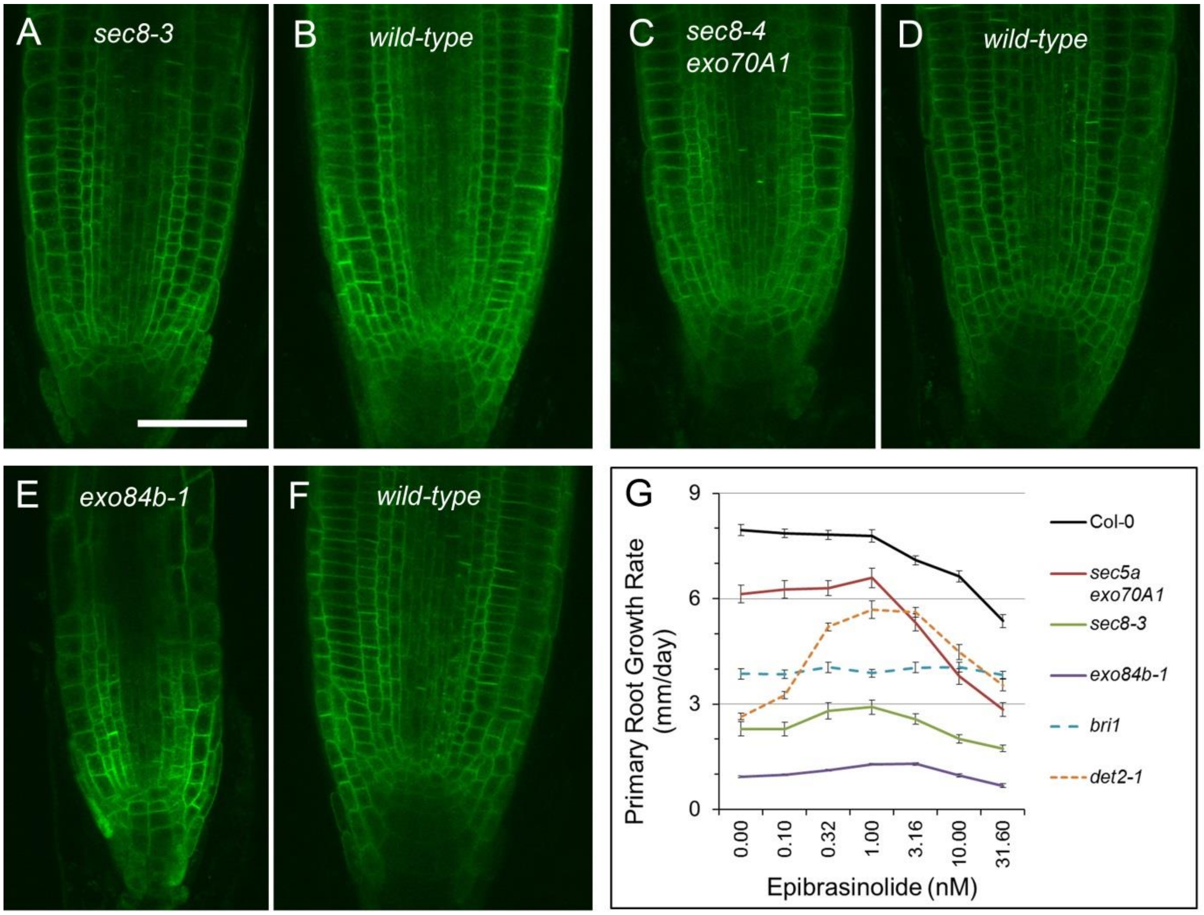

Figure S9. (A-F) BRI1-GFP localization to the periphery (i.e. plasma membrane region) of meristematic cells appears similar in exocyst mutants and their wild-type siblings: *sec8-3* (A) and a wild-type sibling (B); *sec8-4 exo70A1-2* (C) and a wild-type sibling (D); *exo84b-1* (E) and a wild-type sibling (F); bar=50 microns. (G) Primary root growth response to exogenously applied epibrassinolide of exocyst mutants, compared to Col-0, a brassinosteroid signaling mutant (*bri1*, SALK_003371) and a brassinosteroid synthesis mutant (*det2-1*). The exocyst mutants demonstrate a slight rescue of their root growth defect at 1 nM epibrassinolide. Bars=standard error. Normalized growth rate response to epibrassinolide is in Figure 7A of main text.

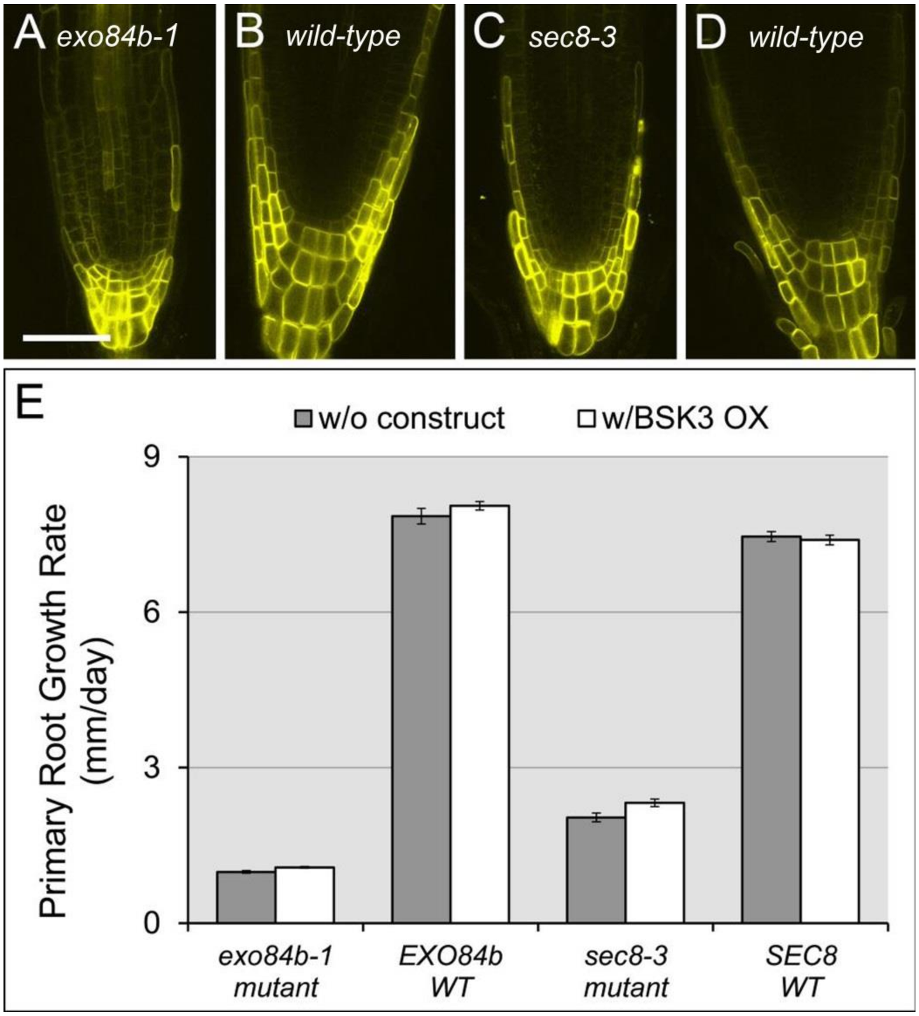

Figure S10. BSK3-YFP over-expression (OX) has a slight effect on exocyst mutant root growth rate. Expression of *p35S:BSK3-YFP* shows similar root tip localization in *exo84b-1* mutants (A) and their wild-type siblings (B), as well as in *sec8-3* mutants (C) and their wild-type siblings (D); bar=50 microns. (E) Over-expression of BSK3 had a slight, but significant, effect: increasing the primary root growth rate in *exo84b-1* ($p<0.01$, t-test, n=41-147) and *sec8-3* mutants ($p<0.01$, t-test, n=54-118), but not in their wild-type siblings ($p>0.1$, t-test, n=30-98); error bars = standard error.

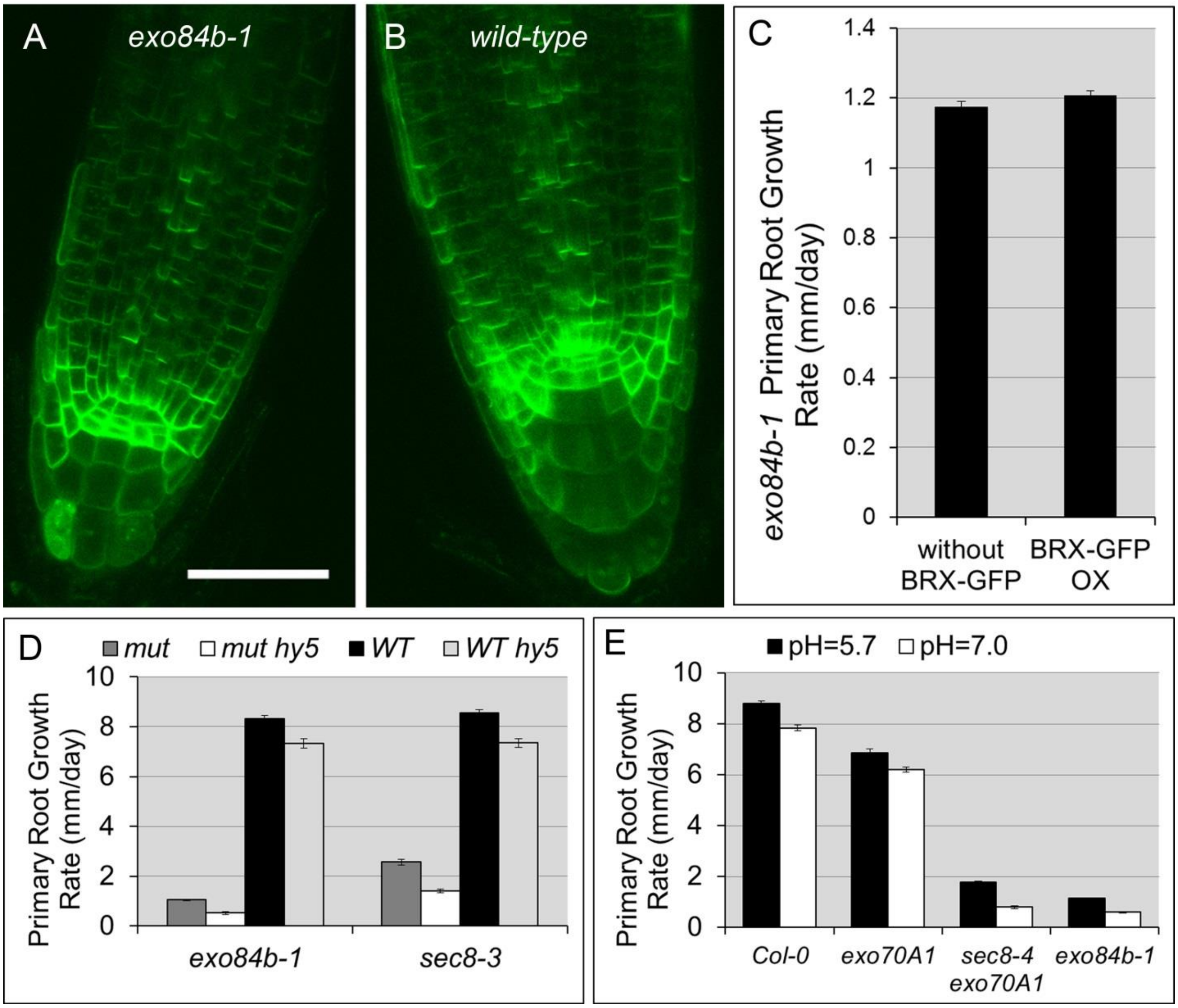

Figure S11. The root growth defect in exocyst mutants is insensitive to manipulations that alter BRX-mediated signaling. Over-expression of BRX (via *p35S:BRX-GFP*) could be visualized under confocal microscopy in the root tips of *exo84b-1* mutants (A) and their wild-type siblings (B), but failed to rescue the primary root growth defect (C) (p>0.05, t-test, n>40); bar = 50 microns. The presence of the *hy5* mutation, which rescues the *brx* mutant root growth defect, failed to rescue the root growth defect in *exo84b-1* or *sec8-3* mutants (D). Growth on a more neutral pH, which also rescues the *brx* phenotype, failed to rescue the root growth defect in exocyst mutants (vs. the standard medium, pH=5.7) (E).
