# Supplementary material for: Developmentally distinct activities of the exocyst enable rapid cell elongation and determine meristem size during primary root growth in Arabidopsis
Source: BMC Plant Biol. 2014 Dec 31;14:386. doi: 10.1186/s12870-014-0386-0 (PMC4302519; doi:10.1186/s12870-014-0386-0)
Supplement: Additional file 2: — Using cortical cell length profiles to compare root growth characteristics. [file 12870_2014_386_MOESM2_ESM.pdf]

Additional File 2:
Using Cortical Cell Length Profiles to Compare Root Growth Characteristics

A constant rate of root growth, reflecting an underlying “steady state” pattern of root cell development, is assumed in the calculations that link root growth, meristem size, and mature cortical cell lengths to cell production rates, cell cycle lengths, and elongation rates. Consequently, seedling were grown on 1x Murashige and Skoog medium, which has been reported to promote steady root growth [1], and the root growth rate and cortical cell length measurements were made seven days after germination. This timing is important because prior to 4-5 days the size of the meristem is still expanding, as auxin-, cytokinin-, and gibberellin-mediated signaling pathways adjust to eventually achieve the mature “steady state” meristem length [2]. Steady root growth was verified in the roots evaluated by comparing growth rate between days 5 and 6 with that occurring between days 6 and 7 (data not shown).

Root cortical cell files were selected for analysis because cortical cells provide an easily identifiable, relatively consistent, and commonly assessed root tissue layer in which the root growth zones are clearly recognizable [3, 4, 5, 6]. The usefulness of measurements of a single tissue layer to interpret overall root growth characteristics arises from the observation that the common longitudinal cell walls between root cell layers constrains the tissues into shared and coordinated rates of cell elongation and division [7, 8]. Consequently, the measurement of the cortical cell files allowed a good comparison of the relative root growth characteristics of various seedling genotypes, but does not necessarily indicate the size of growth zones in all other tissue layers.

When cortical cell lengths are plotted as a function of cell position along the cell file beginning at the quiescent center, distinctive growth characteristics in exocyst mutants are evident (Supp. A-Fig.1A). Rapidly expanding cells are encountered closer to the quiescent center in exocyst mutants compared to wild-type Col 0 roots, reflecting their shorter meristems. In addition, cortical cells expand to a shorter mature cell length in exocyst mutants than in wild type. The cell length profile was used to determine the size of the growth zones (described in the Methods section) as well as several important growth parameters. The cell production rate was calculated by dividing the root growth rate by the average mature cell length [9, 5]. An estimate of the length of the cell cycle was obtained by dividing the number of cells in the meristem by the cell production rate and multiplying by the ln(2) [10], a calculation partially justified by the observation that cells throughout the meristem divide at a near constant rate

[7,10].   This approach identified slower rates of cell division in brassinosteroid mutants, consistent with published results [11, 12] and verifying that the method is capable of detecting prolonged cell cycles.

Also evident in these plots is a high degree of variability in the cell lengths within each individual cell file, which becomes particularly pronounced after cell elongation ceases in the maturation zone of the root, resulting in plots that appear jagged rather than smooth.   Biological variation is also observed in the position in the cell file at which exponential expansion begins, which varies between exocyst genotypes and also within a given genotype (Supp. A-Fig. 1B), although the shape of the plots for a particular genotype appears quite similar.

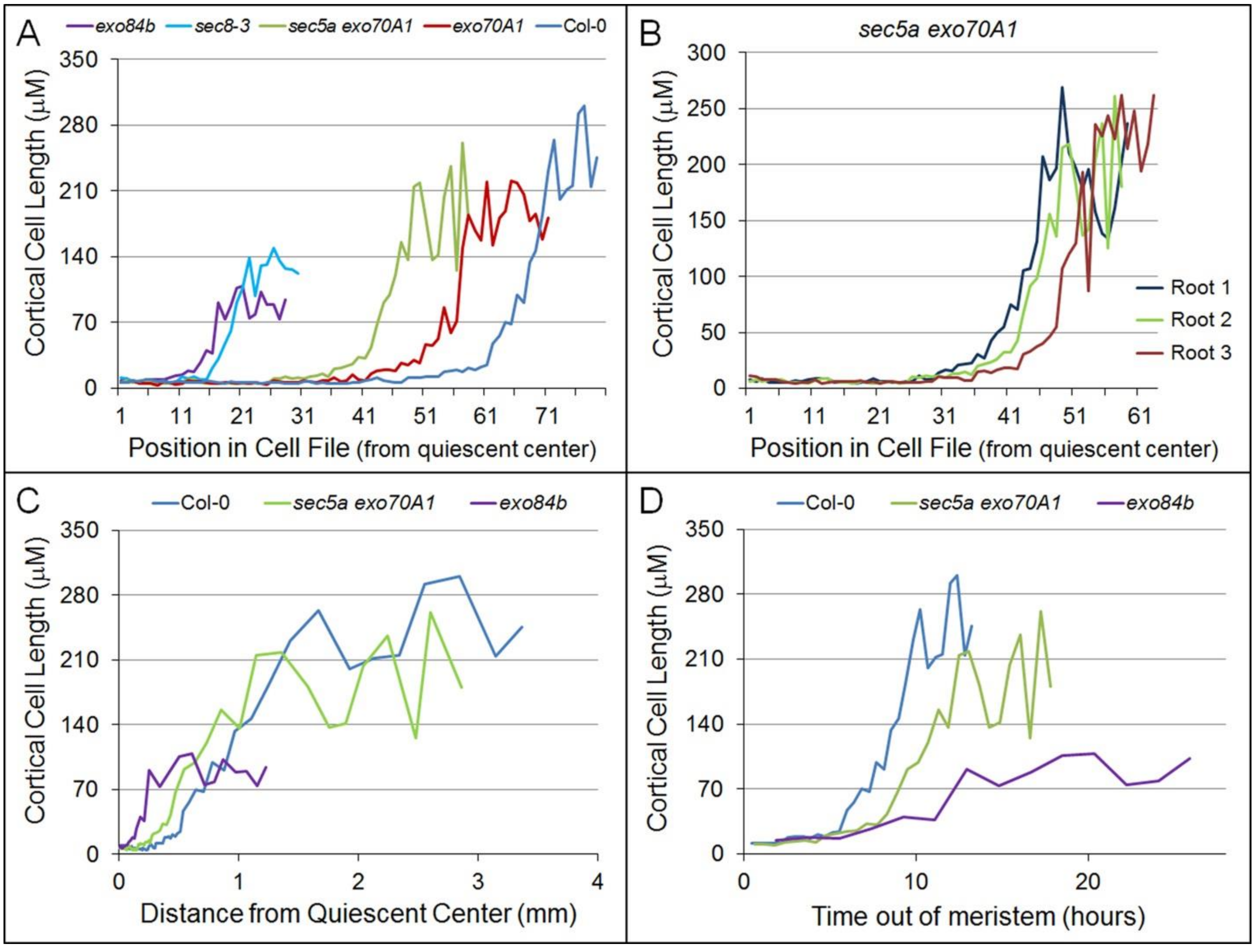

Supplement A - Figure 1. Cell expansion in the elongations zone. (A) Cortical cell length profiles as a function of cell position from single cell files in the root tips of exocyst mutants and Col-0. (B) Cortical cell length profiles as a function of cell position for cell files in three different *sec5a exo70A1* root tips. (C) Cortical cell length profiles as a function of distance from the quiescent center for exo84b, sec5a exo70A1, and Col-0 (using data for the same cell files as

plotted in A). (D) Cortical cell lengths as a function of time out of the meristem (i.e. in the transition/elongation zones) for the same cell files plotted in C.

Cell Elongation in the Elongation Zone

The slope of the curves in the accelerated elongation zone appears roughly similar between all genotypes examined in the plots of cell length as a function of position (Supp. A-Fig. 1A) or as a function of distance from the quiescent center (Supp. A-Fig. 1C). These slopes do not represent the rate of elongation (i.e. the change in cell length as a function of time) because the horizontal axis is not time. One approach to determining the rate of cell elongation involves translating cell position into time by considering the time interval between cortical cells leaving the meristem (i.e. to enter the transition/elongation zone). An estimate of the time interval between consecutive cortical cells “passing” this developmental point is made by taking the inverse of the meristem’s cell production rate [9,13 14]. The time a particular cell has been in the transition/elongation zone can then be estimated from the number of cells that lie between a particular cell and the boundary of the meristem. This allows the cell length data to be plotted as a function of time, as shown in Supp. A Fig. 1D, revealing curves with reduced slopes in exocyst mutants, i.e., the cells in exocyst mutants are expanding at a slower rate compared to wild-type. The validity of this approach depends on constant steady-state root growth, and the absence of cell division in the cell file outside of the meristem. As previously described, care was taken to assure steady state growth in the roots examined. Additionally, while cells were often observed in the process of cell division in the meristem, they were never observed dividing outside of the meristem, nor were there instances outside of the meristem in which two consecutive cells in a cell file had half the length of their neighboring cells, indicative of a recent cell division.

Fourteen cell files (seven roots) for each genotype were evaluated in this way to enable statistical comparisons of cortical cell elongation rates. In this analysis, data for each cell file was taken for that portion of the cell file that was in the elongation zone, specifically identified in each root. This aligned the beginning of the accelerated expansion zones, and thereby adjusted for differences between roots in the cell position at which accelerated cell elongation begins (e.g. differences seen in Supp. A-Fig. 1B). Further, each root’s cell production rate was used to estimate the time interval between cells for that particular root. When the resultant data were plotted with cortical cell length as a function of time (Supp. A-Fig. 2A), the distribution appeared to approximate an exponential function, as has been observed in the elongation zone by others (in supp materials of [13]). Fundamentally, exponential elongation can be described

as $Y = Y_0 e^{kt}$, where “Y” is the length of the cell at time “t”, “Y_0” is the initial length when the cell enters the elongation zone, and “k” is an exponential time constant describing how quickly the cell elongates. Exponential elongation is mathematically predicted if the rate of elongation is proportional to cell length (and also, coincidentally, to the lateral surface of the cell available for exocytosis). Notably, exponential expansion is suggested by a model for cell wall loosening and expansin-mediated cell elongation [15, 16].

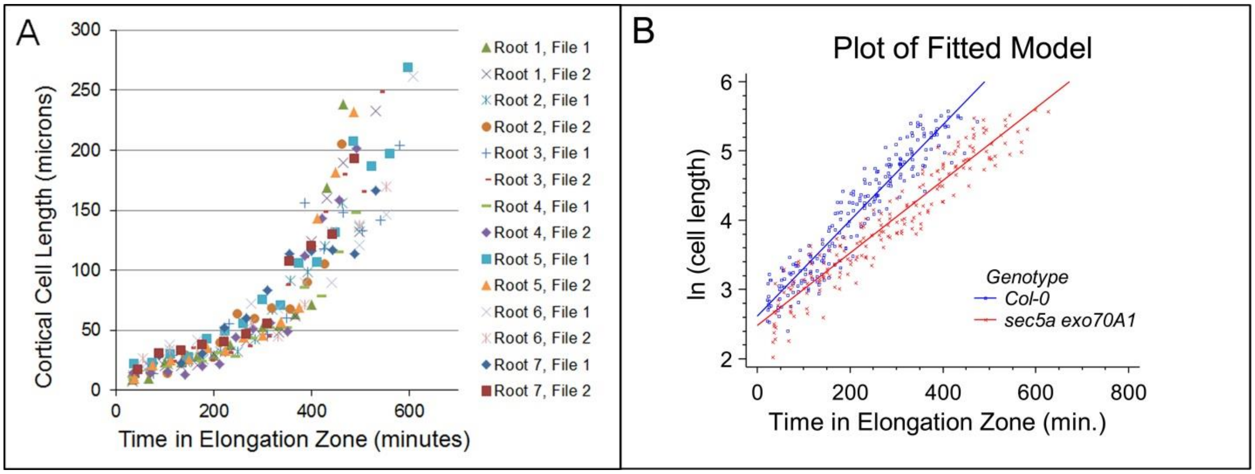

Supplement A – Figure 2. A. Example of cell elongation data as a function of time in the elongation zone for 14 cell files in seven *sec5a exo70A1* roots. B. Cell elongation data for *sec5a exo70A1* (from A) after log transformation and linear regression compared to similar data for Col-0 (STATOGRAPHICS output).

To facilitate comparison of the exponential elongation curves, as well as to determine the closeness-of-fit of the data to an exponential, it was convenient to perform a logarithmic transformation on the cell length data. This transformation converted the exponential function into a linear one, such that $\ln(Y) = \ln(Y_0) + kt$. Linear regression analysis was then used to fit ln(Y) versus time data to a line with a y intercept of $\ln(Y_0)$, and a slope equal to the time constant k. Correlation coefficients and R-squared values indicated a high percentage of variability in the data was explained by the linear relationship Table A1). Differences between the lines generated for different genotypes were then compared for significance using multiple regression / Potthoff analysis [17, 18, 19]. Potthoff analysis was implemented using STATOGRAPHICS Centurion XVI Version 16.1.03 software from StatPoint Technologies, Inc. (An example of STATOGRAPHICS graphical output is Supp. A-Fig. 2B). The exponential rate constants for all mutant lines were significantly different from Col0 ($p<0.001$, Potthoff analysis).

On the other hand, the exponential rate constants for the mutants with the most severe root growth defects (sec8-3; sec8-4 exo70A1-2; and exo84b-1, highlighted in yellow in Table A1) as well as BFA treated Col0 roots were not significantly different from each other ($p>0.05$, Potthoff analysis). The exponential curves describing elongation for each genotype are plotted in Supplement A – Fig. 3, and described in Table A1.

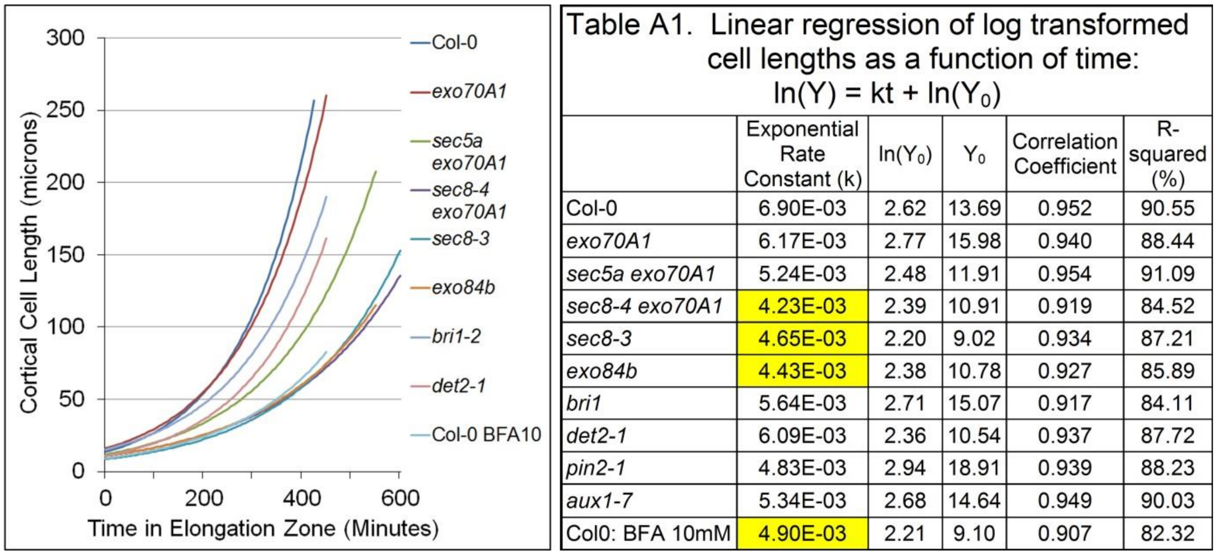

Table A1. Linear regression of log transformed cell lengths as a function of time: $\ln(Y) = kt + \ln(Y_0)$

	Exponential Rate Constant (k)	$\ln(Y_0)$	Y_0	Correlation Coefficient	R-squared (%)
Col-0	6.90E-03	2.62	13.69	0.952	90.55
exo70A1	6.17E-03	2.77	15.98	0.940	88.44
sec5a exo70A1	5.24E-03	2.48	11.91	0.954	91.09
sec8-4 exo70A1	4.23E-03	2.39	10.91	0.919	84.52
sec8-3	4.65E-03	2.20	9.02	0.934	87.21
exo84b	4.43E-03	2.38	10.78	0.927	85.89
bri1	5.64E-03	2.71	15.07	0.917	84.11
det2-1	6.09E-03	2.36	10.54	0.937	87.72
pin2-1	4.83E-03	2.94	18.91	0.939	88.23
aux1-7	5.34E-03	2.68	14.64	0.949	90.03
Col0: BFA 10mM	4.90E-03	2.21	9.10	0.907	82.32

Supplement A – Figure 3. Exponential curves describing cortical cell elongation as a function of time in the elongation zone.

<u>Cell Elongation in the Meristem</u>

Determination of the cortical cell elongation rate in the meristem is more difficult. In the meristem, elongation rates cannot be determined directly from cell length measurements because cells in the meristem are dividing randomly with respect to position in the cell file. Instead we approached the observed cortical cell lengths in the meristem as a probability distribution, and recognized that these cell lengths fall between a maximum length (l_{max}) and ½ of that maximum. We used the 90th percentile of the observed cell lengths as an estimate of l_{max}. After observing root meristematic cell divisions occurring during the course of our confocal microscopy, we noted that the time for cytokinesis is very short relative to the cell cycle length (T). We therefore estimated the rate of elongation as ½ l_{max} divided by T. This is equivalent to assuming a constant rate of elongation from the time the cell divides until just before it divides again. Such a constant rate of elongation would seem to be necessary to accommodate the coordinated elongation between the cell layers comprising the various root tissues where cell division is occurring at different times. Validation of such an assumption was possible by

determining the average length of cells in the meristem. The assumption of a constant rate of elongation predicts that the average cell length will be about 75 percent of I_{max}. If the cell is only elongating for a portion of the cell cycle, then the rate of elongation will need to be higher during that time to achieve the same final length, and the average cell length will deviate from the 75 percent. Analysis of our data showed that the average cell length was close to 75%, varying from 73-83% (Table 2). Furthermore, the results indicate that the cortical root cells of exocyst mutants do not have a slower rate of cell elongation compared to wild-type (Col0), and the assumption of a constant rate of elongation in the meristem appears roughly validated. Importantly, the calculations correctly identify the slower elongation rates known to exist in two brassinosteroid mutants (i.e. *bri1-2* and *det2-1*), validating this approach.

Table A2	Col-0	*exo70A1*	*sec5a exo70A1*	*sec8-4 exo70A1*	*sec8-3*	*exo84b*	*bri1*	*det2-1*	*pin2-1*	*aux1-7*	Col-0 +BFA10
Length of cell cycle (hrs)	14.9	13.3	14.8	13.0	13.3	11.71	20.2	20.1	14.9	14.6	13.1
90th percentile I_{max} (μM)	8.31	8.68	8.41	9.56	10.15	10.59	7.61	6.82	9.80	9.97	10.19
Cell elongation rate (μM/hr)	0.279	0.327	0.285	0.366	0.381	0.452	0.188	0.170	0.329	0.341	0.388
Average cell length (μM)	6.04	6.50	6.73	7.89	7.87	8.61	5.52	5.14	7.53	7.93	7.73
Average as % I_{max}	73%	75%	80%	83%	78%	77%	73%	75%	77%	80%	76%

Evaluation of cell width expansion in the meristem

Confocal images of the meristem were captured in a midline plane that optimized our ability to measure cell lengths in two cortical cell files. These same images were utilized to assess cell widths in the meristem; however, not all cell files were assessed. Both cell files were assessed when the cell widths appeared similar in each file and as might be expected if both were imaged through a midline optical slice. In some roots it was noted that one cell file appeared notably wider than the other, presumably because the optical slice was not through the midline of the narrower cell file. Therefore, to better approximate the true cell widths, data was only collected from the wider cell file in such cases. The resultant sample size was 7-14 cells for each cell position taken from seven roots for each genotype. The plot of the average cortical cell width as a function of cell position for each genotype is provided below (Supp. A – Fig. 4).

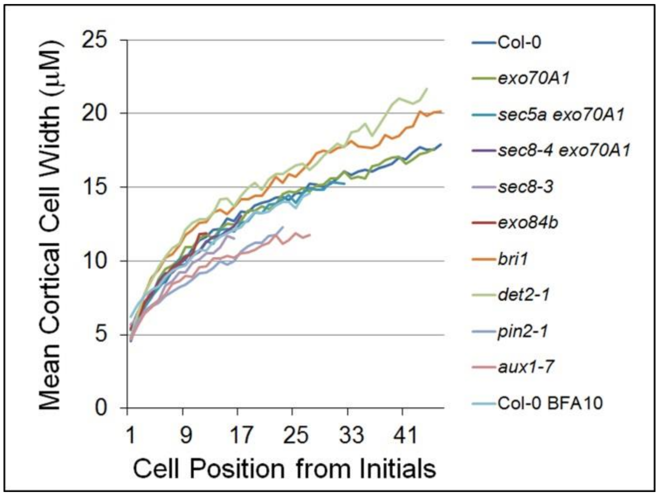

Supp. A – Figure 4. Cell width expansion as a function of cell position in the meristem. Exocyst mutants demonstrate a course of cell width expansion that is similar to Col-0, while auxin mutants (*pin2-1* and *aux1-7*) show slightly less and brassinosteroid mutants (*bri1-2* and *det2-1*) show slightly more cell width expansion compared to Col-0.

References

1 Beemster G, De Vusser K, De Tavernier E, De Bock K, Inze D: **Variation in growth rate between Arabidopsis ecotypes is correlated with cell division and A-Type cyclin-dependent kinase activity**. *Plant Physiol*. 2002, **129**:854-864.

2 Ubeda-Tomás S and Bennett M: **Plant Development: Size Matters, and It's All Down to Hormones**. *Curr. Biol*. 2010, **20**(12): R511-R513.

3 Dolan L, Janmaat K, Willemsen V, Linstead P, Poethig S, Roberts K, Scheres B: **Cellular organization of the Arabidopsis thaliana root**. *Development* 1993, **119**: 71-84.

4 Ioio R, Nakamura K, Moubayidin L, Perilli S, Taniguchi M, Morita M, Aoyama T, Costantino P, Sabatini S: **A Genetic Framework for the Control of Cell Division and Differentiation in the Root Meristem**. *Science* 2008, **322**:1380-1384.

5 Rymen B, Coppens F, Dhondt S, Fiorani F, Beemster G: **Kinematic analysis of cell division and expansion**. In: *Plant Developmental Biology, Methods in Molecular Biology* 2010 **655**: 203-227, Hennic, L, and Kohler, C., eds. (NewYork: Humana Press).

6 Gujas B, Alonso-Blanco C, Hardtke C: **Natural Arabidopsis brx loss-of-function alleles confer root adaptation to acidic soil**. *Curr. Biol*. 2012, **22**:1952-1968.

7 Baskin T: **On the constancy of cell division rate in the root meristem**. *Plant Mol. Biol.* 2000, **43**: 545-554.

8 Ivanov V, Dobrochaev A, Baskin T: **What the distribution of cell lengths in the root meristem does and does not reveal about cell division**. *J. Plant Growth Regul*. 2002, **21**: 60-67.

9 Silk W, Lord E, Eckard K: **Growth patterns inferred from anatomical records: empirical tests using longisections of roots in Zea mays L**. *Plant Physiol*. 1989, 90(2): 708-713.

10 Ivanov V, Dubrovsky J: **Estimation of the cell-cycle duration in the root apical meristem: a model of linkage between cell-cycle duration, rate of cell production, and rate of root growth**. *Int. J. Plant Sci*. 1997, **158**(6): 757-763.

11 González-García M, Vilarrasa-Blasi J, Zhiponova M, Divol F, Mora-García S, Russinova E, Caño-Delgado A: **Brassinosteroids control meristem size by promoting cell cycle progression in *Arabidopsis* roots**. *Development* 2011,**138**: 849-859

12 Hacham Y, Holland N, Butterfield C, Ubeda-Tomás S, Bennett M, Chory J, Savaldi-Goldstein S: **Brassinosteroid perception in the epidermis controls root meristem size**. *Development* 2011, **138**: 839-848.

13 Band L, Ubeda-Tomás S, Dyson R, Middleton A, Hodgman T, Owen M, Jensen O, Bennett, M., and King, J: **Growth-induced hormone dilution can explain the dynamics of plant root cell elongation**. *PNAS* 2012, **109**(19): 7577-7582.

14 Ron M, Dorrity M, de Lucas M, Toal T, Hernandez R, Little S, Maloof J, Kliebensten D, Brady S: **Identification of novel loci regulating interspecific variation in root morphology and cellular development in Tomato**. *Plant Physiol*. 2013, **162**: 755-768.

15 Pietruszka M: **Exact analytic solutions for a global equation of plant cell growth**. *J. Theor. Biol*. 2010, **264**:457-466.

16 Pietruszka M: **Solutions for a local equation of anisotropic plant cell growth: an analytical study of expansin activity**. *J. R. Soc*. 2011, Interface **8**: 975-987

17 Potthoff R F: **Statistical aspects of the problem of bias in psychological tests**. *Institute of Statistics Mimeo Series 0.479*, 1978, Chapel Hill: University of North Carolina, Department of Statistics.

18 Watkins M, Hetrick C: **MacPotthoff: automated calculation of the Potthoff regression bias procedure.** *Behav. Res. Meth. Instrum. Comput*. 1999, **31**(4): 710-711.

19 Wuensch, K. **Comparing regression lines from independent samples**. Retrieved from http://core.ecu.edu/psyc/wuenschk/MV/MultReg/Potthoff.pdf on June 1, 2013.
