# Supplementary material for: Developmentally distinct activities of the exocyst enable rapid cell elongation and determine meristem size during primary root growth in Arabidopsis
Source: BMC Plant Biol. 2014 Dec 31;14:386. doi: 10.1186/s12870-014-0386-0 (PMC4302519; doi:10.1186/s12870-014-0386-0)
Supplement: Additional file 3: — qRT-PCR primers. [file 12870_2014_386_MOESM3_ESM.pdf]

Additional File 3:

Primers for expression analysis by qRT-PCR

DWF4 (At3g50660), *CPD* (At5g05690), *TIP41* (At4g34270), *SAND* (At2g28390), and expressed protein (At4g33380) were selected for expression analysis by qRT-PCR. DWF4 and CPD are involved in brassinosteroid biosynthesis, and their expression is subject to feedback inhibition, i.e. their expression is down-regulated by elevated brassinolide [1, 2, 3, 4]. *TIP41*, *SAND*, and At4g33380 were selected as reference genes based upon their documented stability of expression and successful use in qRT-PCR studies of roots [5, 6, 7, 8, 9]. Primers used, along with qRT-PCR efficiencies and R^2 values determined from serial dilution calibration curves are as follows:

Gene	Primer	Amplicon Size (bases)	bp from 3' end of cDNA	Efficiency	R^2
CPD<br>NM_001036761	CAGGCCCTTCTAATGTGTTCA<br>CCCAACTG^AAGCCTGTCACTA	120	314	96.6%	0.995
DWF<br>NM_114926	CATCGCAAAGCACTCAAAGAT<br>TTATCCAAATGTACGGCTGAGA	95	514	97.8%	0.975
TIP41<br>NM_119592	GC^GAAAGGGTATCCAGTTGA<br>GGAAGCCTCTGACTGATGGA	65	343	97.3%	0.995
SAND<br>NM_128399	TGATCCACTTGCAGACA^AGG<br>TACCCTTTGGCACACCTGAT	61	310	99.0%	0.990
Expressed Protein<br>NM_119492	TGTTGGCTTAAATAC^GCAGAGA<br>TGCTCAAGCCGTTACAACAC	129	265	96.4%	0.976
^ signifies location of intron in genomic DNA that is spanned by this primer for cDNA					

References

1 Nemhauser J, Chory J: **BRing in on: new insights into the mechanism of brassinosteroid action.** *J. Exp. Bot*. 2004, **55**(395): 265-270.

2 Goda H, Sawa S, Asami T, Fujioka S, Shimada Y, Yoshida S: **Comprehensive comparison of auxin-regulated and brassinosteroid-regulated genes in Arabidopsis**. *Plant Physiol*. 2004, **134**: 1555-1573.

3 Tanaka K, Asami T, Yoshida S, Nakamura Y, Matsuo T, Okamoto S: **Brassinosteroid homeostasis in Arabidopsis is ensured by feedback expressions of multiple genes involved in its metabolism**. *Plant Physiol*. 2005, **138**(2): 1117-1125.

4 Yoshimitsu Y, Tanaka K, Fukuda W, Asami T, Yoshida S, Hayashi K, Kamiya Y, Jikumaru Y, Shigeta T, Nakamura Y, Matsuo T, Okamoto S: **Transcription of**

***DWARF4* plays a crucial role in auxin-regulated root elongation in addition to brassinosteroid homeostasis in *Arabidopsis thaliana*.** *PLoS ONE* 2011, **6**(8): e23851.

5 Czechowski T, Stitt M, Altmann T, Udvardi M, Scheible W: **Genome-wide identification and testing of superior reference genes for transcript normalization in Arabidopsis.** *Plant Physiol*. 2005, **139**: 5-17.

6 Expósito-Rodríguez M, Borges A, Borges-Pérez A, Pérez J: **Selection of internal control genes for quantitative real-time RT-PCR studies during tomato development process.** *BMC Plant Biol*. 2008, **8**:131

7 Remans T, Smeets K, Opdenakker K, Mathijsen D, Vangronsveld J, Cuypers A: **Normalization of real-time RT-PCR gene expression measurements in Arabidopsis thaliana exposed to increased metal concentrations**. *Planta* 2008, **227**: 1343-1349.

8 Rieu I, Eriksson S, Powers S, Gong F, Griffiths J, Woolley L, Benlloch R, Nilsson O, Thomas S, Hedden P, Phillips A: **Genetic analysis reveals that C19-GA 2-oxidation is a major gibberllin inactivation pathway in Arabidopsis**. *Plant Cell* 2008, **20**: 2420-2436.

9 Tromas A, Braun N, Muller P, Khodus T, Paponov I, Palme K, Ljung K, Lee J, Benfey P, Murray J, Scheres B, Perrot-Rechenmann C: **The AUXIN BINDING PROTEIN 1 is required for differential auxin responses mediating root growth**. *PLos ONE* 2009, **4**(9):e6648.
